# The Influence of Above-Ground Herbivory on the Response of Arctic Soil Methanotrophs to Increasing CH_4_ Concentrations and Temperatures

**DOI:** 10.3390/microorganisms9102080

**Published:** 2021-10-02

**Authors:** Edda M. Rainer, Christophe V. W. Seppey, Caroline Hammer, Mette M. Svenning, Alexander T. Tveit

**Affiliations:** 1 Department of Arctic and Marine Biology, UiT, The Arctic University of Norway, 9037 Tromsø, Norway; seppey@uni-potsdam.de (C.V.W.S.); c.hammer@boku.ac.at (C.H.); mette.svenning@uit.no (M.M.S.); alexander.t.tveit@uit.no (A.T.T.); 2 Institute of Environmental Sciences and Geography, University of Potsdam, Karl-Liebknecht-Str. 24-25, 14476 Potsdam, Germany

**Keywords:** methanotroph, methane oxidation, pmoA amplicon sequencing, *Methylobacter*, grazing pressure, peat soil microcosms, temperature, Arctic

## Abstract

Rising temperatures in the Arctic affect soil microorganisms, herbivores, and peatland vegetation, thus directly and indirectly influencing microbial CH_4_ production. It is not currently known how methanotrophs in Arctic peat respond to combined changes in temperature, CH_4_ concentration, and vegetation. We studied methanotroph responses to temperature and CH_4_ concentration in peat exposed to herbivory and protected by exclosures. The methanotroph activity was assessed by CH_4_ oxidation rate measurements using peat soil microcosms and a pure culture of *Methylobacter tundripaludum* SV96, qPCR, and sequencing of pmoA transcripts. Elevated CH_4_ concentrations led to higher CH_4_ oxidation rates both in grazed and exclosed peat soils, but the strongest response was observed in grazed peat soils. Furthermore, the relative transcriptional activities of different methanotroph community members were affected by the CH_4_ concentrations. While transcriptional responses to low CH_4_ concentrations were more prevalent in grazed peat soils, responses to high CH_4_ concentrations were more prevalent in exclosed peat soils. We observed no significant methanotroph responses to increasing temperatures. We conclude that methanotroph communities in these peat soils respond to changes in the CH_4_ concentration depending on their previous exposure to grazing. This “conditioning” influences which strains will thrive and, therefore, determines the function of the methanotroph community.

## 1. Introduction

Vegetation in parts of the Arctic has changed severely due to increased grazing by geese [1,2,3]. The higher geese numbers are closely linked to climate and land-use changes, as food sources are increasing in their overwintering grounds [4,5]. Herbivory-induced changes in vegetation lead to changes in below-ground biota and their productivity [6,7,8] and affect the balance of greenhouse gases in wetlands, with higher methane (CH_4_) emissions from areas subjected to grazing [8,9,10,11]. The exclusion of herbivores from patches of Arctic peatlands led to the restoration of vascular plant growth [12], and it was shown that higher abundances of vascular plants alter the polysaccharide composition in these soils, further changing the soil microbial community [13]. 

At the same time, the Arctic is warming two to three times faster than the global average, an effect known as Arctic amplification [14,15,16]. A result of this is the acceleration of permafrost thaw and a thickening of the active layer, with longer periods of non-frozen topsoil [17]. Thawing permafrost contains organic carbon, which can be metabolized by microbes that emit carbon dioxide (CO_2_) and CH_4_. It has been shown that even small increases in temperatures can lead to substantial increases in CH_4_ production by peat soil microorganisms under anoxic conditions [18,19,20,21]. Increased CH_4_ production can potentially lead to increased CH_4_ emissions, further increasing global temperatures through radiative forcing.

CH_4_ production is counterbalanced by methane-oxidizing bacteria (MOB; methanotrophs). MOB act as a biological filter for CH_4_ and play a key role in mitigating CH_4_ emissions from peat and other soils. Many MOB live at the oxic/anoxic interface in peatland soils [22,23], oxidizing CH_4_ for energy harvest and biomass synthesis. MOB are a diverse group of bacteria belonging to the phyla *Gamma-* and *Alphaproteobacteria* and *Verrucomicrobia* [24]. The diversity of MOB communities is affected by environmental variables such as pH and oxygen (O_2_). Many cold and neutral pH soils are inhabited by low-diversity communities of MOB, dominated by the genus *Methylobacter,* e.g., [25,26,27]. In contrast, cold acidic soils are more likely to be dominated by members of the *Alphaproteobacteria*, such as the genera *Methylocystis* and *Methylocapsa* [24,28,29,30].

MOB found in Arctic soils have temperature growth patterns ranging from psychrotrophic to mesophilic; these MOB communities can, therefore, respond to large temperature changes [26,31,32,33]. However, physiological and ecological temperature responses and how these affect community CH_4_ oxidation rates are not well understood. In line with our limited understanding of this, studies have shown that temperature effects on CH_4_ oxidation rates are inconsistent, ranging from, for example, strong temperature responses in landfill soils [34] to variable responses in permafrost soils [35] and a lack of responses in forest soils [36]. On the contrary, the physiological responses of different MOB species to temperature based only on growth are highly predictable [37]. The relationship between temperature, growth rates, and soil CH_4_ oxidation rates depends on numerous variables, such as soil CH_4_ and nutrient concentrations, enzyme kinetics, physiological acclimation, and growth responses of individual strains and overall MOB population sizes. Additionally, the types and numbers of different strains responsible for oxidation are also likely to affect CH_4_ oxidation rates.

Due to the numerous controls on MOB communities and their activities, it may be anticipated that the type of soil habitat strongly influences potential CH_4_ oxidation rates. In adjacent but chemically and structurally different permafrost soil habitats, different CH_4_ oxidation rates were observed, with higher rates in soils exposed to the highest CH_4_ concentrations [35]. Substrate availability, that is, CH_4_ concentrations, have been shown to control CH_4_ oxidation rates in both lakes and forest soils [38,39]. Similarly, peat exposed to herbivory displayed differently structured microbiota, including altered MOB habitats and higher CH_4_ oxidation potentials than peat protected from herbivory by barnacle geese [13,27].

However, it is still unclear how peat MOB communities respond to changes in temperature and CH_4_ production. It is also unknown how herbivory influences this response. The objective of this study was to investigate the effect of increasing CH_4_ concentrations and temperatures on MOB community activities in samples representing two different Arctic peat soil ecosystem states—(1) peat exposed to geese grazing for more than 20 years, and (2) peat protected from grazing for the last 20 years.

## 2. Materials and Methods

### 2.1. Field Site and Sampling

Samples were collected in August 2016 at Solvatn. The Solvatn peatland (N78°55.550, E11°56.611) is located close to the Ny Ålesund research station and settlement in Svalbard. It is heavily grazed by Barnacle geese (*Branta leucopsis*) and dominated by brown mosses, primarily *Calliergon richardsonii* [40]. Exclosures established in 1998 protect parts of the peatland vegetation from grazing geese [12], allowing for the growth of vascular plants that are otherwise suppressed by grazing. From one site, named SV1 in a recent study [27], two blocks (approximately 30 × 15 × 30 cm) were cut from the peat soil protected from grazing (SV1 EX1 and SV1 EX2) as well as from the adjacent grazed peat soils (SV1 GR1 and SV1 GR2) and directly transferred to zip-lock plastic bags. All four blocks were kept cool during transportation from the field sites to the on-site laboratory (approximately 5 min) and then frozen at −20 °C upon arrival. The blocks were further shipped, frozen, to the laboratory in Tromsø, Norway, and stored at −20 °C until being used for the microcosm experiments. The experiments were not carried out in the field laboratory due to lacking infrastructure. Consequently, the samples were stored frozen to avoid extended storage in a non-frozen condition that could lead to system changes. These soils are exposed to freezing conditions for most of the year, including freeze and thaw occurrences during spring and autumn. Nevertheless, to evaluate the effects of freezing, CH_4_ oxidation rates were quantified both immediately after thawing and again after a three-week incubation. We also added an additional pre-incubation period to one of our two experiments to test whether prolonged incubation would lead to even higher oxidation rates. Details of this step are presented in Section 2.2.

### 2.2. Soil Microcosms

This study was based on two peat soil microcosm experiments, referred to as Exp I and Exp II. The microcosms were organized in a fully crossed factor design to study the combined effect of two CH_4_ concentrations and two temperatures on the two grazing treatments (grazed and exclosed peat).

The zone of maximal CH_4_ oxidation in the Arctic peat soil, as previously identified in situ [27], was used for the experiments. In grazed soils, this zone was located at a depth of 0.5–2.0 cm, while in exclosed soils, it was located at a depth of 4–8 cm [27].

The experimental temperatures were 8 °C and 15 °C. The lower temperature of 8 °C is a frequently encountered surface soil temperature at this high latitude during summer (5.4–10.0 °C for grazed soils and 4.4–9.8 °C for exclosed soils at a depth of 5 cm, [27]), while 15 °C is at the high end of temperatures recorded in the vegetation layer of Arctic peat soil in summer [27,41,42]. The experimental headspace CH_4_ concentrations were 0.1% (about 2 µM of dissolved CH_4_ at 8 °C and 1.7 µM of dissolved of CH_4_ at 15 °C) and 1% (about 20 µM of dissolved CH_4_ at 8 °C and about 17 µM of dissolved CH_4_ at 15 °C), imitating commonly occurring CH_4_ concentrations in the upper layers of these soils. Soil CH_4_ concentrations previously observed ranged from 100.7–237.4 µM (avg 153.5 µM) in grazed sites at a depth of 5 cm and from 0.4–3.7 µM (avg 1.5 µM) in exclosed sites at a depth of 10 cm [27]. 

For Exp I, peat blocks were thawed at 8 °C overnight. Layers 0–2 cm deep of the grazed peat soil and layers 5–7 cm deep of the exclosed peat soil were cut from the peat blocks. Then, 4 g of peat soil were weighed into 50 mL serum bottles (16 bottles with grazed and 16 bottles with exclosed peat soil). Each vial was closed with butyl rubber stoppers (Wheaton) and aluminum crimp caps [43].

We injected 0.07 mL CH_4_ (95% *v/v*) into 16 bottles (8 bottles per grazing treatment) to obtain a headspace concentration of 0.1% CH_4_, while 16 bottles (8 bottles per grazing treatment) were injected with 0.7 mL CH_4_ (95% *v/v*) for a headspace concentration of 1% CH_4_. For each grazing treatment, four bottles with 0.1% CH_4_ and four bottles with 1% CH_4_ were incubated at 8 °C and at 15 °C, respectively. In total, the experiment consisted of 32 bottles.

At the start of the experiment, the initial CH_4_ oxidation rates were calculated from four CH_4_ concentration measurements made within the first 20–40 h for each bottle. At each time point, 0.5 mL gas samples were retrieved from the headspace with a pressure-lock syringe (Vici Precision Sampling, LA, USA) and injected directly onto a Haysep-D packed column of a GC-FID (SRI Instruments, CA, USA). The elution time was 1.8 min for CH_4_, and the instrument was set to maximum sensitivity. The CH_4_ concentrations were calculated by comparing them to the injected standard gases (Messer, Switzerland). After the first four sampling time-points, an incubation period of three weeks started, during which the CH_4_ headspace concentrations were maintained at 0.1% or 1%. The CH_4_ was consumed rapidly in some of the microcosms and was, therefore, supplied at regular intervals to ensure that the concentrations never dropped below 0.05% or 0.8% CH_4_ for the 0.1% or 1% conditions, respectively.

At the end of the incubation period, the CH_4_ oxidation rates were estimated with the four measurements described above for the start of the experiment. After the CH_4_ oxidation rate measurements, the peat soil from each microcosm was transferred into sterile 15 mL tubes and flash frozen in liquid N_2_ to be used for RNA extraction.

After evaluating the results of the first experiment, we decided to perform a second experiment (Exp II) with a slightly altered setup, designed to provide even more certainty on three potentially crucial factors—soil depth, pre-incubation length, and pre-incubation temperature.

Twice the number of microcosm replicates for all conditions (64 bottles in total) were used in Exp II to better represent the variability in potential CH_4_ oxidation rates between the two grazing treatments. We also included a pre-incubation week at 4 °C for all microcosms. For the 15 °C microcosms, this was followed by another pre-incubation week at 8 °C to avoid abrupt large temperature increases unlikely to occur in nature. Because of this additional week, the microcosms incubated at 15 °C were incubated for one week longer than the 8 °C microcosms, while the incubation at the final temperature (i.e., 8 or 15 °C) was the same for both sets of microcosms. In Exp II, we targeted a soil layer 4 cm thick to ensure that the entire zone of the highest CH_4_ oxidation potential [27] was included. The considerable variation in the CH_4_ oxidation rates between some replicates in Exp I suggest that the selection of a narrow 2 cm layer could have excluded the most active zone, in some cases, due to local variations. Therefore, we sampled a layer at a depth of 0–4 cm for the grazed treatment and at a depth of 4–8 cm for the exclosed treatment for Exp II. The bottles were then prepared in the same way as for Exp I.

The soil water content was determined gravimetrically by drying 5 g of soil from each layer at 150 °C in triplicate. The determined water content was used to calculate the soil dry weight and the CH_4_ oxidation rate per gram of dry soil for each microcosm.

To consider the different solubility of CH_4_ in pore water at 8 °C and 15 °C, the CH_4_ oxidation rates for the same dissolved concentrations of CH_4_ were estimated. Assuming first-order rate kinetics, as previously shown in [44], we log-transformed the decline in CH_4_ over time and fitted linear regression models to the transformed plots. The slopes of the linear models correspond to the rate constants. By multiplying the respective rate constants by a CH_4_ concentration that would result in the same dissolved CH_4_ concentrations at 8 and 15 °C, we obtained adjusted rates at these temperatures. The fit of the linear model was evaluated and considered to have a satisfactory coefficient of determination, giving the following values: R^2^—8 °C = 0.71 to 0.99, R^2^—15 °C = 0.71 to 0.99; 147 of the 192 linear models had an R^2^ of > 0.9.

### 2.3. CH_4_ Oxidation of Methylobacter Tundripaludum SV96

*M. tundripaludum* SV96 was cultivated in NMS medium [45] for 10–14 days at 8 and 15 °C under a headspace CH_4_ concentration of approximately 13% (20 mL of a mixture of 95% CH_4_ and 5% CO_2_). To reach exponential growth, the culture was transferred to fresh medium three times during this period. Serum bottles were incubated on rotary shakers at 150 rpm. Exponentially growing cells were harvested and diluted in sterile 0.5 mM phosphate buffer (pH = 6.8) to a density of about 5 × 10^7^ cells per ml. Then, 21.6 mL of this diluted cell suspension was distributed into 125 mL serum bottles (3 bottles per concentration and temperature; 24 in total), which were then closed with butyl rubber stoppers (Wheaton) and aluminum crimp caps. Headspace atmospheres were prepared by injecting different amounts of CH_4_ (0.2, 0.6, 1.5, and 3 mL of 95% CH_4_ and 5% CO_2_) to create a series of CH_4_ headspace concentrations (about 1900, 5400, 13,000, and 25,000 ppm, respectively) with three bottles per CH_4_ concentration. In addition, a negative control that contained only phosphate buffer and no bacterial cells was measured.

The CH_4_ concentrations were measured at four time points (including time point zero, right after the headspace preparation) over the course of eight hours. To measure the headspace CH_4_ concentration, 0.5 mL of gas was collected with a pressure-lock syringe (VICI) and directly injected into a GC-FID, as was described for the soil microcosms. Starting at time point one, 340 µL of cell suspension was collected from each of the bottles after the CH_4_ measurements to estimate the cell density. The cell density was measured by optical density (OD) at 410 nm. The densities were converted into cell numbers with a standard of cell numbers and OD measurements specifically made for *M. tundripaludum* SV96. The standard was established by cultivating *M. tundripaludum* SV96 to 14 different optical densities ranging from 0.0085 OD410 to 0.7295 OD410 and counting, for each OD, the number of cells at that optical density using a counting chamber under a light microscope. The cell counts ranged from 6.1 to 726.6 × 10^5^, corresponding to a linear model R^2^ of 0.92. The estimation of cell numbers allowed us to adjust the CH_4_ oxidation rates to the number of cells as this number increased throughout the experiment. Between measurements, the flasks were incubated at the respective temperatures on a rotary shaker at 150 rpm [46].

The headspace and dissolved CH_4_ concentrations were calculated from the measured peak areas using a range of defined standards. Concentrations were converted into masses of CH_4_ using the ideal gas law, while Henry’s law was used to estimate the corresponding mass of dissolved CH_4_. All numbers were adjusted for the removal of gases and medium during the sampling. The CH_4_ oxidation rates calculated between each time point were then normalized to the time between measurements and the number of cells in the medium to obtain a rate (µmol of CH_4_ oxidized per 10^8^ cells per hour).

Assuming first-order rate kinetics, as previously shown in [44], we log-transformed the cell-normalized decline in CH_4_ over time and fitted linear regression models to the transformed plots. The slopes of the linear models correspond to the rate constants. By multiplying the respective rate constant by the desired concentration of CH_4_, we obtained the rates of oxidation at these concentrations [44]. The fit of the linear models was evaluated and considered to have satisfactory coefficients of determination, giving the following values: R^2^—8 °C = 0.83–0.99, R^2^—15 °C = 0.72–0.99; 24 of the 28 linear models had an R^2^ of > 0.9.

### 2.4. RNA Extraction

Nucleic acids were extracted from the peat soil samples from Exp I. The samples were homogenized using a mortar and pestle while suspended in liquid nitrogen. The nucleic acids were extracted from 0.2 g of peat soil (two technical replicates per sample) using a modified version of Griffiths’ protocol [42,47]. For RNA extraction, the two replicates were pooled, and DNA was removed (RQ1 DNase, Promega, Madison, WI, USA), which was followed by RNA clean-Up (MegaClear^TM^ Transcription Clean-Up Kit, Ambion^TM^, Thermo Fisher Scientific, Waltham, MA, USA) and ethanol precipitation. The RNA quality was controlled by gel electrophoresis and a Nanodrop (Thermo Fisher Scientific, Waltham, MA, USA). The RNA was reverse transcribed using Superscript IV (Thermo Fisher Scientific, Waltham, MA, USA) and the cDNA was used as a template for PCR with the primer pair A189F/mb661R [48] to confirm the presence of the *pmoA* gene transcript. The cDNA samples were sent for amplicon generation and sequencing with Illumina MiSeq at the IMGM laboratories in Planegg, Germany. For the amplicon generation, both the A189F/mb661 and A189F/A682R primers sets were applied, further referred to as the mb661R and A682R datasets [48,49]. The *pmoA* transcript amplicons were generated in a 2-step target-specific (TS) PCR (using 10 ng of cDNA as a template) for 25 cycles, followed by a 12-cycle index PCR using 1 μL from the TS PCR products. The polymerase used for amplification was the Q5^®^ High-Fidelity polymerase from NEB (Ipswich, MA, USA).

### 2.5. PmoA Transcript Numbers

The cDNA samples were diluted to 2.5 ng/µL for quantification with qPCR. For the standard curve, genomic DNA of *M. tundripaludum* SV96 was checked for integrity using gel electrophoresis and quantified using Qubit Fluorometric Quantification (Thermo Fischer Scientific, Waltham, MA, USA). Copy numbers of *pmoA* were calculated based on genomic information and the measured concentration. The standard curve was generated using dilutions of the DNA from *M. tundripaludum* SV96, yielding 7.15 × 10^6^ to 7.15 *pmoA* copies. The diluted cDNA samples and the diluted standards were analyzed in triplicate. The negative controls (no template) were analyzed simultaneously in at least six replicates. The master mix and primer for the qPCR were the SsoFast Evergreen Supermix (Biorad, Hercules, CA, USA) and the A189/mb661R primer pair [48], respectively. The thermal profile for qPCR amplification [50] was used with the following modifications: Fluorescence data acquisition was performed at 78 °C for 30 s, and a total of 35 cycles were performed after the initial 10 cycles of touchdown qPCR. Melting curve analysis was performed from 65 °C to 95 °C. The calculations for each replicate were performed within the qPCR software (CFX Manager, Biorad, Hercules, CA, USA) based on the standard curve. The number of *pmoA* transcripts per gram of dry soil was calculated based on the average of the three replicates.

### 2.6. Bioinformatics

#### 2.6.1. Databases

For the taxonomical assignation, a de-replicated database was built with sequences trimmed according to the corresponding *pmoA* primer sets. Sequences used for the two *pmoA* databases [51] were complemented with three *Methylobacter* sequences (GenBank id=AJ414658.1, KC878619.1, and the sequence from an Arctic coalmine isolate from Svalbard (not published)).

#### 2.6.2. Pipeline

Reads were merged using the program Flash (v. 1.2.8; [52]) and low-quality sequences were discarded if they had a 50 nucleotide fragment with an average Phred score below 20 prior to trimming the primers. Chimeras were removed using the program Vsearch (v. 2.4.4; [53]), both against a database (for *pmoA* primers [51]) and de novo. To avoid frameshift errors, only sequences without stop codon errors or framing errors (the nucleotide number is not divisible by three) were kept. OTU clustering was performed using Swarm (v. 2.1.13; [54]), and taxonomically assigned using the best alignment between the dominant sequence of each OTU and the database, using Ggsearch36 (v. 36.3.8f; [55]). Finally, the OTUs were selected according to their dominant sequence length (mb661: (465,474)), A682: (492,495)) to remove obvious sequencing errors. An overview of all the steps in the pipeline is given in Supplementary Table S1.

#### 2.6.3. Community Analyses

To normalize for sequencing depth, we used the relative abundance of the two MOB communities. To avoid noise caused by low-abundance OTUs in a given sample, every OTU representing less than a thousandth of the community in this sample was considered as absent from that sample. This denoising step removed less than 5% of the reads.

#### 2.6.4. Redundancy Analysis (RDA)

Prior to analyses, the community matrices were log-normalized according to the methods of [56] (function decostand, package vegan v. 2.4-2; [57]). For each primer pair, the effects of elevated CH_4_ and elevated temperature were estimated for the microbial communities using a redundancy analysis (RDA, function capscale, package vegan v. 2.4-2; [57]). To only consider the effect of the CH_4_ and temperature treatments, the effects of the peat block replicates were considered random variables. The effect of the factors, as well as the significance of the ordination axes were tested with a permutation test (10,000 permutations, function anova.cca, package vegan v. 2.4-2; [57]). To disentangle the effects of interactions between the grazing treatments, an RDA was calculated for each grazing treatment and tested in the same fashion as the analysis performed on all samples.

#### 2.6.5. Bioindicators

For each primer pair and grazing treatment, the “responding bioindicator OTUs” for both CH_4_ concentrations were identified using an indicator species analysis (indval; function indval, package labdsv v. 1.8-0; [58]). An OTU was selected as a “responding bioindicator OTU” if the probability of reaching a higher indicator value than that of the bioindicator after 10,000 permutations was lower than 0.01. “Responding bioindicator OTUs” were only reported in heatmaps.

We further selected the “unconditional bioindicator OTUs” by one of two criteria—(1) OTUs that respond to either 0.1% or 1% CH_4_ in both the grazed and exclosed peat soil microcosms; (2) OTUs that respond to either 0.1% or 1% CH_4_ in the grazed peat soil and are absent from exclosed peat soil microcosms, or the other way around.

#### 2.6.6. Phylogenetic Analysis

For the phylogenetic analysis, only “unconditional bioindicator OTUs” were included. Phylogenetic trees were then built from the “unconditional bioindicator OTU“ sequences and closely related sequences were retrieved from the NCBI GenBank to better assess their taxonomy. These closely related sequences were obtained by aligning the bioindicator sequences (using BLASTn) against the NCBI nucleotide database and selecting the two-highest scoring matches. A set of cultivated gammaproteobacterial MOB sequences were retrieved in addition to a set of *pmoA* sequences belonging to upland-soil cluster (USC)-gamma, the latter serving as an outgroup to root the tree. Sequences were aligned in MEGA10 [59] using MUSCLE with UPGMB clustering [60]. The length of the alignment was inspected visually for an overlap in all sequences and a section of 452 bp (the mb661R dataset) was chosen for phylogenetic analysis. A phylogenetic tree was constructed in MEGA10 using the neighbor-joining method with the Jukes–Cantor correction and 500 bootstraps [59]. In addition, phylogenetic trees using the maximum likelihood and the minimum evolution method (with the Jukes–Cantor correction and 500 bootstraps each) were generated and compared to the first tree.

## 3. Results

### 3.1. CH_4_ Oxidation Rates Changing in Response to CH_4_ Concentrations

**Figure 1:**
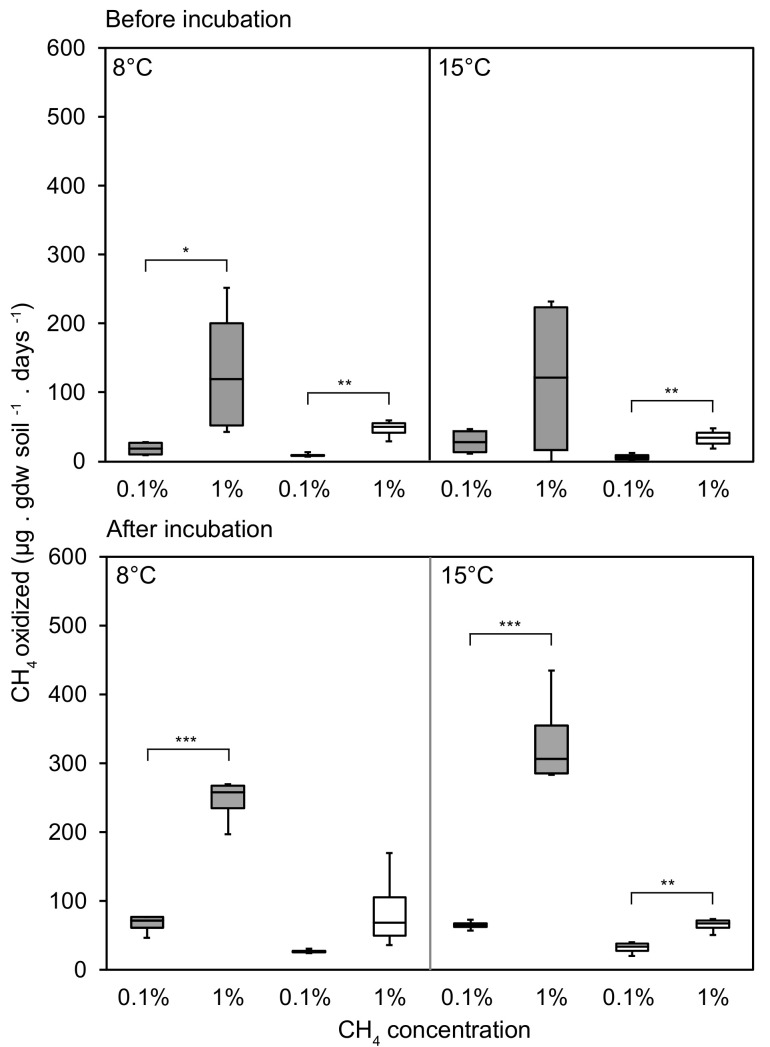
CH_4_ oxidation rates from Exp I, emphasizing the comparison between 0.1% and 1% headspace CH_4_ concentrations. Results are shown for measurements collected before (top) and after (bottom) the three-week incubation period, and at 8 (left) and 15 °C (right). Grazed microcosms are shown in gray; exclosed microcosms in white. Comparison using linear mixed models with peat blocks as random variables shows significant differences in the CH_4_ oxidation rates between CH_4_ concentrations (* *p* < 0.05; ** *p* < 0.01; *** *p* < 0.001).

Incubation under microcosm headspace atmospheres with 1% CH_4_ resulted in higher CH_4_ oxidation rates than with 0.1% CH_4_ for both grazed and exclosed peat soils. This effect was observed both before and after the three-week incubation period (Figure 1 and Supplementary Figure S1). Additionally, the CH_4_ oxidation rates were always higher after the three-week incubation (Supplementary Figure S2 and Figure S3), showing that the MOB community responded positively to prolonged incubation at stable conditions after thawing. However, the oxidation rates after the three-week incubation period were similar in Exp I (Supplementary Figure S2) and Exp II (Supplementary Figure S3), confirming that the additional pre-incubation at 4 °C and 8 °C in Exp II had negligible effects on the CH_4_ oxidation rate. This suggests that within the first three weeks, this MOB community had reached its full potential under the present conditions. Overall, higher CH_4_ oxidation rates were observed in the grazed peat soils.

### 3.2. CH_4_ Oxidation in Response to Temperature

**Figure 2:**
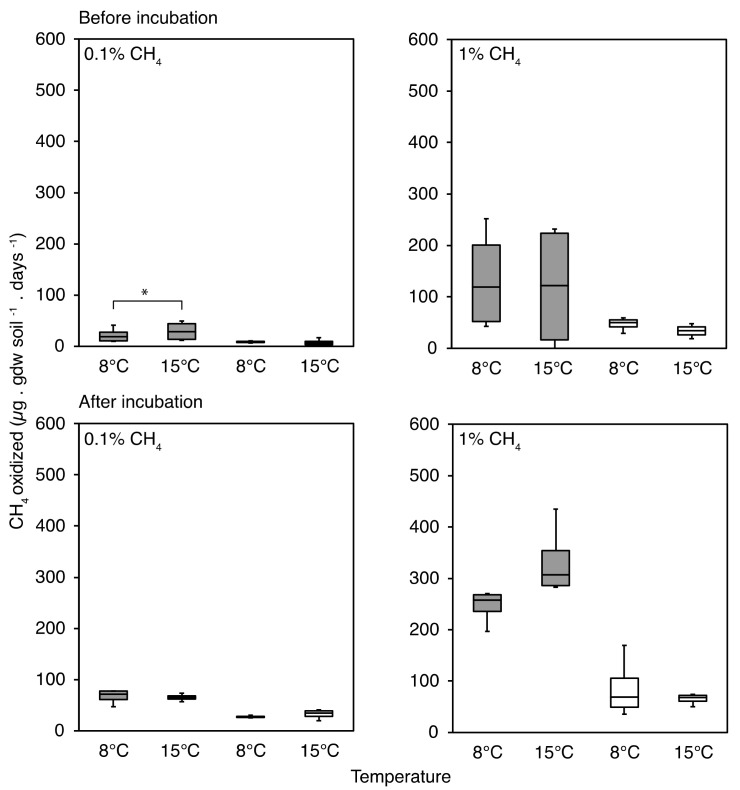
CH_4_ oxidation rates from Exp I, emphasizing the comparison between 8 °C and 15 °C. Results are shown for measurements collected before (top) and after (bottom) the three-week incubation period, and at 0.1% (left) and 1% CH_4_ (right). Grazed microcosms are shown in gray; exclosed microcosms in white. Comparison using linear mixed models with peat blocks as random variables shows significant differences in the CH_4_ oxidation rates between temperatures (* *p* = 0.05–0.01).

We observed mostly minor and insignificant temperature effects on CH_4_ oxidation rates in grazed and exclosed peat soils, both before and after the three-week incubation (Figure 2 and Supplementary Figure S4). Exceptions to this were the significantly higher CH_4_ oxidation rates at 15 °C compared to 8 °C in grazed peat soils at 0.1% CH_4_ before the three-week incubation in both experiments. In exclosed peat soils from Exp II, significantly higher CH_4_ oxidation rates at 15 °C were also measured at 1% CH_4_ before the incubation, while significantly lower rates were measured in exclosed peat soil microcosms at 0.1% CH_4_ after the incubation.

To identify whether these inconsistencies and sometimes a lack of temperature effects on CH_4_ oxidation rates were due to the reduced solubility of CH_4_ with increasing temperatures (i.e., less CH_4_ dissolved with a given headspace concentration at 15 °C than at 8 °C), we estimated the rate for the same dissolved concentration of CH_4_ at both temperatures, assuming first-order rate kinetics (see Section 2.2 for details). This showed that even when accounting for the lower solubility of CH_4_ at 15 °C, we found no consistent significant differences in the rates between 8 °C and 15 °C (Supplementary Figure S5 and Figure S6).

### 3.3. CH_4_ Oxidation in a Pure Culture of M. Tundripaludum SV96 in Response to Temperature

**Figure 3:**
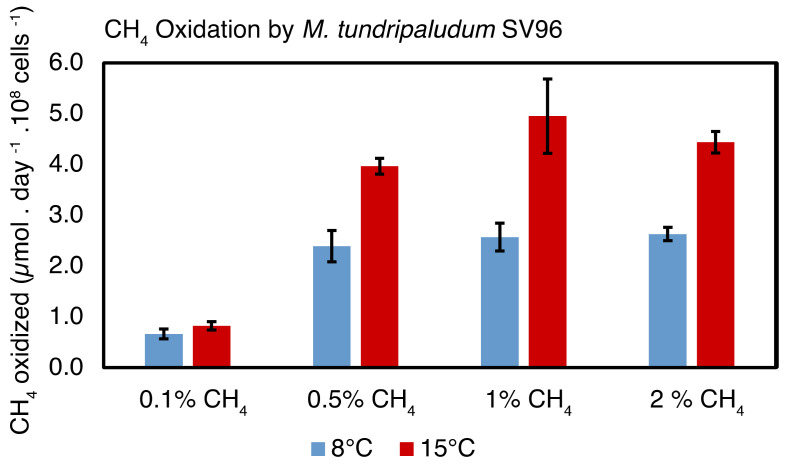
CH_4_ oxidation per cell for *Methylobacter tundripaludum* SV96 at 8 °C (blue) and 15 °C (red) with CH_4_ headspace concentrations ranging from 0.1% to 2%.

*M. tundripaludum* SV96 was isolated from the peat soil habitat sampled for the experiments above [32]. To better understand the soil oxidation rates and temperature responses described above, we designed a CH_4_ oxidation experiment with *M. tundripaludum* SV96 using the same two temperatures (8 °C and 15 °C) and CH_4_ concentrations (0.1% and 1%) for comparison. We observed the highest CH_4_ oxidation rates per cell number at 1% CH_4_ concentration and we also observed a clear effect of temperature at this concentration, with approximately twice as high CH_4_ oxidation rates at 15 °C compared to 8 °C (Figure 3).

However, the temperature effect on the CH_4_ oxidation rate at 0.1% headspace concentration was much smaller and not significant (*p* = 0.3). Adjusting the rate according to the CH_4_ solubility, we observed a similarly small but significant difference (*p* = 0.006, Supplementary Figure S7).

### 3.4. MOB Community

#### 3.4.1. *PmoA* Transcript Numbers

**Figure 4:**
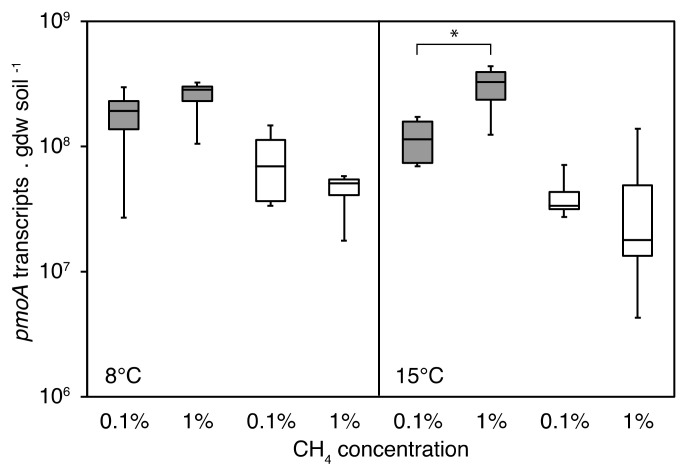
MOB *pmoA* transcript number after the three-week incubation at 0.1% and 1% CH_4_ in grazed (gray) and exclosed sites (white). The left panel shows microcosms incubated at 8 °C and the right panel shows microcosms incubated at 15 °C. The y-axis shows *pmoA* transcript numbers per gram dry soil, log-transformed. Pairwise comparison using linear mixed models with peat blocks as random variables shows significant differences in the *pmoA* transcripts (* *p* = 0.05–0.01).

A higher number of *pmoA* transcripts at 1% CH_4_ than at 0.1% CH_4_ was detected in the grazed peat soils. This difference was significant at 15 °C (linear mixed model (lmm) *p* = 0.04), but not at 8 °C (lmm *p* = 0.28) (Figure 4). In the exclosed peat soils, no significant differences in *pmoA* transcript numbers related to the CH_4_ concentration were detected. Overall, the *pmoA* transcript numbers were lower for the exclosed peat soils than for the grazed peat soils (0.1% CH_4_ lmm *p* = 0.04 at 15 °C; 1% CH_4_ lmm *p* = 0.01 at 8 °C, and *p* = 0.02 at 15 °C). An exception was found at 0.1% CH_4_ at 8 °C (lmm *p* = 0.07), suggesting a transcriptionally less active MOB community was responsible for the lower CH_4_ oxidation rates in the exclosed peat soils.

#### 3.4.2. Transcriptionally Active MOB

**Figure 5:**
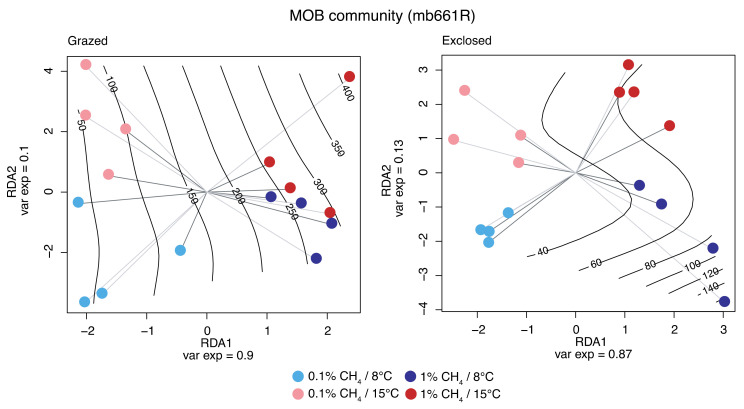
Redundancy analysis of the effect of CH_4_ concentration and temperature on the MOB communities after incubation (*pmoA* transcripts using the A189F/mb661R primer pair). The effect of CH_4_ concentration and temperature was corrected according to the peat block replicates (represented by gray shaded lines). Samples are labeled according to CH_4_ concentration (light colors—0.1% CH_4_, dark colors—1% CH_4_) and temperature (blue—8 °C and red—15 °C). The black lines indicate a projection of the measured CH_4_ oxidation rates (µg of CH_4_ oxidized per gram dry soil and day).

The active MOB communities were identified by sequencing *pmoA* transcripts from Exp I. The CH_4_ concentration had a significant effect on the composition of MOB community transcripts (*p* < 0.001 for both the mb661R and the A682R dataset) (Figure 5 and Supplementary Figure S8). Temperature, on the other hand, had no significant effect on the MOB community transcript composition (*p* = 0.264 for mb661R and *p* = 0.449 for A682R datasets).

**Figure 6:**
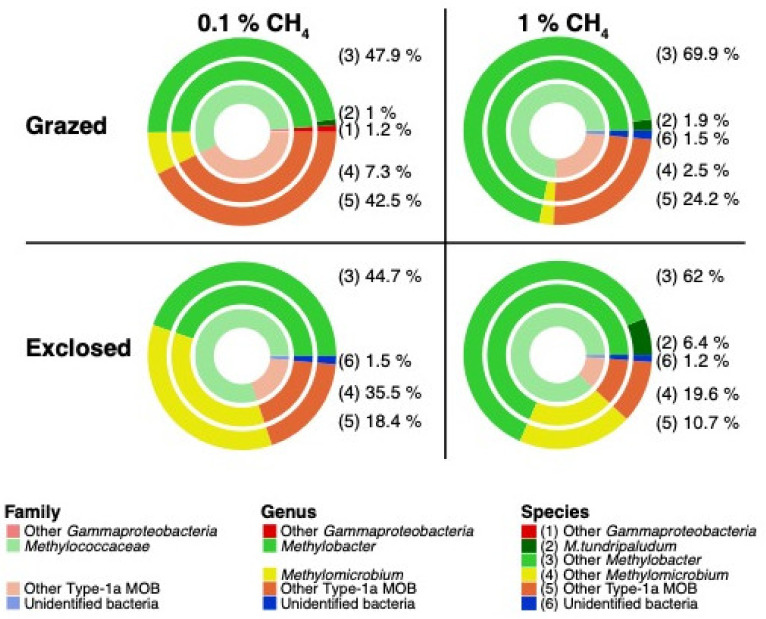
Distribution of MOB taxa (A189F/mb661R primer set) in grazed and exclosed peat soils after incubation at 0.1% CH_4_ and at 1% CH_4_ (16 soil samples per concentration and 16 soil samples per grazing condition). As temperature had no significant effect on the MOB community, these 16 samples include both the 8 and 15 °C incubations. OTU clustering was performed using Swarm [54] and taxonomically assigned by using the best alignment between the dominant sequence of each OTU and the database using Ggsearch36 [55]. Taxa with a relative abundance of > 1% are represented at the full taxonomic resolution, whereas those taxa not reaching the 1% threshold were added to their broader taxonomic group, one level lower in taxonomic resolution. The different rings represent different taxonomic levels from family to genus to species, moving from the innermost ring outward. Relative abundances for the species level are given in %.

The majority of the detected MOB *pmoA* transcripts in both the mb661R and the A682R dataset belonged to the family *Methylococcacaea* (Figure 6 and Supplementary Figure S9). However, the samples incubated at 0.1% CH_4_ contained smaller fractions of *Methylococcacaea* than the samples incubated at 1% CH_4_ (Figure 6 and Supplementary Figure S9).

*Methylobacter* OTUs dominated the 0.1% CH_4_ incubations with average relative abundances of 48.9% (grazed soils) and 44.7% (exclosed soils) (Figure 6, mb661R). In the 1% CH_4_ incubations, *Methylobacter* OTUs made up even larger fractions, with abundances of 71.8% (grazed soils) and 68.4% (exclosed soils) (Figure 6). Contrary to *Methylobacter*, OTUs within *Methylomicrobium* and unclassified Type 1a MOB OTUs had higher relative abundances in the 0.1% CH_4_ incubations than in the 1% CH_4_ incubations.

Similar observations were made in the A682R dataset (Supplementary Figure S9). The lowest relative abundances of *Methylobacter pmoA* transcripts were observed at 0.1% CH_4_, making up 60.9% (grazed soils) and 72.9% (exclosed soils). In the 1% CH_4_ incubations, *Methylobacter* OTUs made up larger fractions of 75.9% (grazed soils) and 79.9% (exclosed soils). *Methylosarcina pmoA* transcripts, which were not detected in the mb661R dataset, had the highest relative abundances in the 0.1% CH_4_ incubations, with 37.6% (grazed soils) and 12.3% (exclosed soils). At 1% CH_4_, these OTUs made up only 21.8% (grazed soils) and 7.7% (exclosed soils).

In the 0.1% CH_4_ incubations, the relative abundances of OTUs matching *M. tundripaludum* SV96 were 1.0% (grazed soils) and <1.0% (exclosed soils) (Figure 6, mb661R dataset). In the 1% CH_4_ incubations, the relative abundances were higher, at 1.9% (grazed soils) and 6.4% (exclosed soils). The same patterns were observed in the A682R dataset (Supplementary Figure S9), with <1.0% (grazed and exclosed soils) and >4.2% (grazed and exclosed soils) at 1% CH_4_. This shows that *M. tundripaludum* SV96 is a small but active part of the MOB community in these soils at high CH_4_ concentrations.

To identify the MOB OTUs that were preferentially activated by either 0.1% or 1% CH_4_, we screened our datasets for OTUs that were consistently more abundant at one or the other CH_4_ concentration and called them “responsive bioindicator OTUs”. From those OTUs, we selected the OTUs that behaved the same in the grazing treatments (grazed and exclosed peat soil) or that were only present in one of the treatments and called those “unconditional bioindicator OTUs” (see Section 2.6.5. for details about the bioindicator selection).

**Figure 7:**
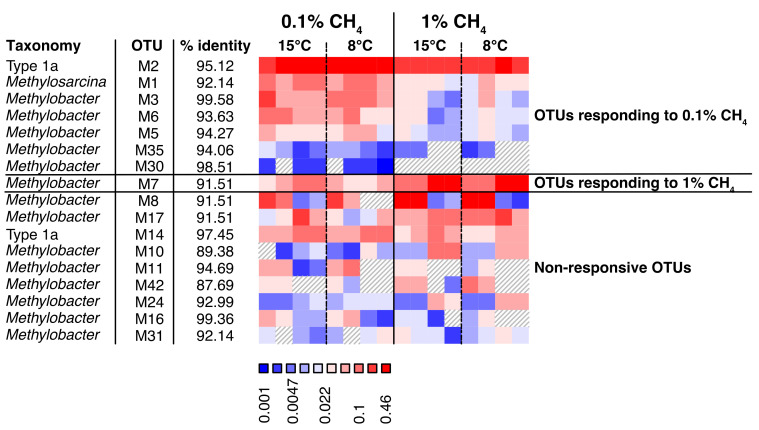
Relative abundances of *pmoA* transcripts representing MOB OTUs (A189F/mb661R primer pair) from grazed peat soil microcosms after incubation at 0.1% and 1% CH_4_ concentrations. OTUs with higher relative abundance at 0.1% CH_4_ are shown in the first block (left), and those with higher relative abundance at 1% CH_4_ in the second block (right). The non-responsive OTUs with the highest relative abundance are shown in the third block. The number of OTUs included in the figure make up 90% of the total MOB community transcription. OTUs are identified by the letter M (indicating that these are part of the mb661R dataset) and a number. The percentage of identity between the OTU and the best hit in the database is given next to the OTU identification. The highest possible taxonomic rank identified was chosen for each OTU. Relative abundances reach from low abundances (blue) to high (red).

**Figure 8:**
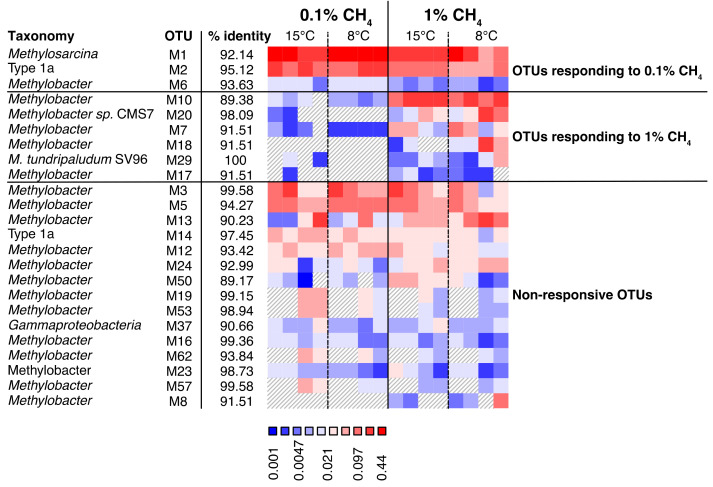
Relative abundances of *pmoA* transcripts representing MOB OTUs (A189F/mb661R primer pair) from exclosed peat soil microcosms after incubation at 0.1% and 1% CH_4_ concentrations. OTUs with higher relative abundance at 0.1% CH_4_ are shown in the first block (left) and those with higher relative abundance at 1% CH_4_ in the second block (right). The non-responsive OTUs with the highest relative abundance are shown in the third block. The number of OTUs included in the figure make up 90% of the total MOB community transcription. OTUs are identified by the letter M (indicating that these are part of the mb661R dataset) and a number. The percentage of identity between the OTU and the best hit in the database is given next to the OTU identification. The highest possible taxonomic rank identified was chosen for each OTU. Relative abundances reach from low abundances (blue) to high (red).

In the grazed soils, we identified 12 “responsive bioindicator OTUs”. Seven (mb661R) and three (A682R) OTUs had higher relative abundances at 0.1% CH_4_, and one OTU in each of the datasets (mb661R and A682R) had a higher relative abundance at 1% CH_4_ (Figure 7, Figure 8 and Supplementary Figure S10 and Figure S11). In the exclosed peat soil microcosms, we identified 15 “responsive bioindicator OTUs”. Three (mb661R) and two (A682R) OTUs had higher relative abundances at 0.1% CH_4_, whereas six (mb661R) and four (A682R) OTUs had a higher relative abundance of transcripts at 1% CH_4_ (Figure 7, Figure 8 and Supplementary Figure S10 and Figure S11).

Considering the two datasets, primed by mb661R and A682R, two OTUs had the highest relative abundances at 1% CH_4_ in both grazed and exclosed soils, while five OTUs had the highest relative abundances at 1% CH_4_ in exclosed soils and were only found in exclosed soils. These OTUs were considered “unconditional bioindicator OTUs” for 1% CH_4_. Four OTUs had the highest relative abundance at 0.1% CH_4_ in both grazed and exclosed peat soils, while three OTUs had the highest relative abundance at 0.1% CH_4_ in either grazed or exclosed peat soil microcosms but were not present in the other soil type. These OTUs were, therefore, considered “unconditional bioindicator OTUs” for 0.1% CH_4_.

**Figure 9:**
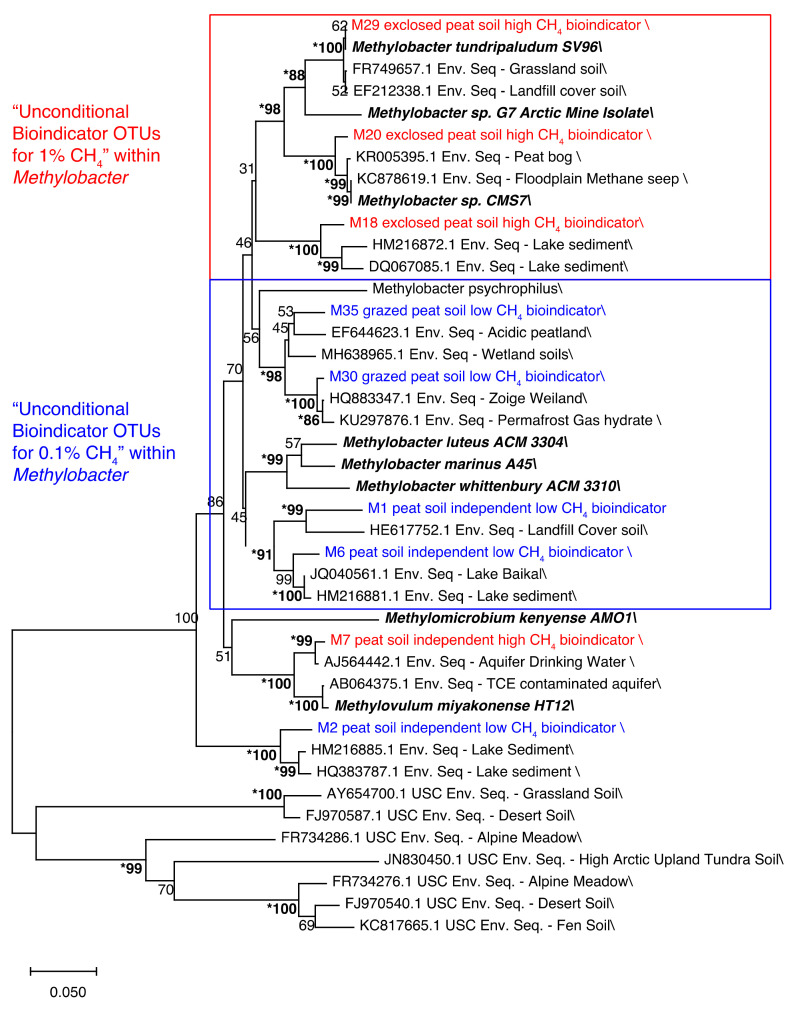
Phylogenetic representation of the “unconditional bioindicator OTUs” for 0.1% CH_4_ (blue) and 1% CH_4_ (red) incubations (mb661R dataset), cultivated MOB (bold, italic), and closely related environmental sequences retrieved from the NCBI GenBank. All OTUs included were identified as bioindicators both in the grazed and the exclosed peat soil microcosms, or in only one of the soil types, being absent from the other soil type. The first type is called “peat soil independent”. The other type of OTUs, which were specifically assigned as bioindicators for one of the peat soil types, are labeled as either grazed or exclosed. The tree is based on a 456-nucleotide alignment, using the neighbor-joining method with the Jukes–cantor correction and 500 bootstraps. The length of the branches is based on a scale of 0.05 changes per nucleotide. Bootstrap values of >80 in 3 models that were used to construct the tree (neighbor-joining, maximum likelihood, and minimum evolution) are marked with * and written in bold.

The *pmoA* sequences categorized as “unconditional bioindicator OTUs” were subsequently aligned with a set of reference sequences, including blast search hits and cultivated strains, and used to infer the phylogenetic relationships of the “unconditional bioindicator OTUs” from the mb661R and A682R datasets, respectively (Figure 9 and Supplementary Figure S12). The *Methylobacter* OTUs identified as “unconditional bioindicator OTUs” for 0.1% CH_4_ clustered separately from those that were “unconditional bioindicator OTUs” for 1% CH_4_ (Figure 9 and Supplementary Figure S12). The two Arctic strains, *M. tundripaludum* SV96 [32] and *Methylobacter* sp. CMS7 [31], clustered with two of the 1% CH_4_ bioindicator OTUs, respectively (Figure 9), whereas the remaining bioindicator OTUs clustered with environmental sequences (including lake sediments and landfill cover soils rather than cultivated strains).

## 4. Discussion

### 4.1. CH_4_ Response

#### 4.1.1. Activity

Higher CH_4_ concentrations led to higher CH_4_ oxidation rates immediately after addition, but the effect was stronger after a three-week incubation period at maintained CH_4_ concentrations. Thus, while the initial rates reflected the oxidation potential of the MOB community shortly after thawing, the latter rates were influenced by MOB growth, physiological acclimation, or community response. Responses of the MOB communities to increasing CH_4_ concentrations were shown in a study of lake overturn, where population shifts over several months corresponded with the ability of the MOB community to mitigate the majority of the CH_4_ [61]. In the same study, a growth response was also indicated in the MOB community, which was responsible for the oxidation of the CH_4_. Such an effect of CH_4_ concentrations on the MOB response and capacity was also shown in a study of atmospheric CH_4_ oxidation by conventional MOB, where conditioning with high CH_4_ concentrations allowed for atmospheric CH_4_ uptake [62]. The highest CH_4_ oxidation rates and strongest response after the three-week incubation in our experiment were observed in grazed peat soil microcosms. These soils contained in situ CH_4_ concentrations of up to 237.4 μM (100.7–237.4 µM of dissolved CH_4_ at SV1) in the pore water at 5 cm and 2139.0 μM (469.4–2139.0 µM dissolved CH_4_) at a depth of 10 cm [27]. In comparison, exclosed peat soils contained only up to 3.7 µM of CH_4_ (0.4–3.7 µM CH_4_ at SV1) at a depth of 10 cm and 578.0 µM (0.53–578 µM of CH_4_) at a depth of 15 cm. Thus, the 0.1% incubations (with a concentration of about 1.7–2 µM of dissolved CH_4_) were lower than in situ CH_4_ concentrations in grazed peat at depths of 5 and 10 cm and about in situ CH_4_ concentrations in exclosed soils at a depth of 10 cm. The 1% incubations (with a concentration of about 17–20 µM of dissolved CH_4_) were lower than the in situ CH_4_ concentrations in grazed soils below 5 cm, whereas they were higher than in situ CH_4_ concentrations of exclosed soils at a depth of 10 cm. It should be noted that the samples for our experiments were collected from the surface (Exp I: 0–2 cm, Exp II: 0–4 cm) in grazed soils and above 10 cm (Exp I: 5–7 cm, Exp II: 4–8 cm). Thus, the microorganisms in these hotspots for CH_4_ oxidation would have been exposed to lower CH_4_ concentrations than indicated by the available in situ pore water CH_4_ measurements. Measurements at 0–5 and 0–10 cm in the grazed and exclosed sites, respectively, were not possible due to the lack of pore water at these depths.

Nevertheless, these results show that the MOB responses were related to the dissolved CH_4_ concentrations in the respective soil pore waters, with stronger MOB responses to added CH_4_ in the soils with higher in situ CH_4_ concentrations.

#### 4.1.2. MOB Transcript Numbers

We observed that a transcriptionally more active MOB community was responsible for the higher CH_4_ oxidation rates in the grazed peat soils compared to the exclosed peat soils. Positive correlations between *pmoA* transcript numbers and CH_4_ oxidation rates have been shown before [23,63]. However, within each grazing treatment, we did not observe a correlation between *pmoA* transcript numbers and CH_4_ oxidation rates. Reasons for this may be changes in population sizes or changes in particulate methane monooxygenase kinetics due to a shift in the MOB community.

#### 4.1.3. MOB Transcript Composition

CH_4_ concentration had a significant impact on the relative abundance of *pmoA* transcripts for different members of the MOB community in both grazed and exclosed peat soils (Figure 5). These results corroborate previous results, suggesting that in situ CH_4_ concentrations shape the MOB community in the Solvatn peatland [27]. The relative abundances of *Methylobacter* OTUs were higher at a 1% CH_4_ concentration in both the grazed and exclosed plots (Figure 6 and Supplementary Figure S9). In contrast to this, there was a lower relative abundance of *Methylomicrobium* and *Methylosarcina* OTU transcripts at a 1% CH_4_ concentration in both the grazed and exclosed plots. *Methylobacter* has been shown to thrive in colder, CH_4_-rich, environments with a neutral pH, and our study confirms that this genus thrives when CH_4_ concentrations are high.

Furthermore, we identified a strong treatment-dependent community transcriptional response, with some OTUs responding positively to 1% CH_4_ (the majority of reacting OTUs were *Methylobacter*), while others responded to 0.1% CH_4_ (Figure 7, Figure 8 and Supplementary Figure S10 and Figure S11). Interestingly, a larger number of OTUs responded to 0.1% CH_4_ in the grazed peat soil microcosms, where in situ CH_4_ concentrations were the highest, while more OTUs responded to 1% CH_4_ in the exclosed peat soil microcosms, which had the lowest in situ CH_4_ concentrations [27]. This suggests that high and low in situ CH_4_ concentrations select for different MOB strains, respectively, and that the largest group of strains respond to the condition that is most different from the in situ condition. We suspect that this is because the responding strains had low transcriptional activity in the original soil and became more active during steady exposure to a 0.1% CH_4_ headspace concentration. Furthermore, our findings show that such effects are observable within three weeks and correlate with changes in the CH_4_ oxidation rates. This indicates the co-existence of closely related strains that prefer either high or low CH_4_ concentrations, respectively, and that these populations can replace each other within an Arctic summer. Similar observations were made in lakes, where changes in apparent half-saturation constants and CH_4_ uptake rates at the CH_4_ saturation correlated with changes in *pmoA* expression and for which MOB strains (99% identity) were responsible [61]. It is tempting to draw a line between which strains are active and use this as a basis for inferring whether the strains have high or low apparent affinities for CH_4_. However, cell numbers and cellular enzyme contents also influence uptake rates, and the relative contribution of enzyme kinetics, cell numbers, and cellular protein content to the observed rates remains unknown in most studies, including our own. Nevertheless, the different CH_4_ uptake rates seem to correspond with different compositions of active MOB strains.

#### 4.1.4. Phylogeny

Most of the bioindicators belonged to the genus *Methylobacter*. Some of the “unconditional bioindicator OTUs” for 1% CH_4_ clustered with high-Arctic *Methylobacter* isolates retrieved from CH_4_-rich ecosystems [31,32], while the phylogenetic clusters containing the “unconditional bioindicator OTUs” for 0.1% CH_4_ did not contain closely related isolates. Atmospheric MOB are phylogenetically distinct but closely related to conventional MOB [24,64] and are characterized by a more efficient energy metabolism [44]. Similarly, phylogenetically different populations of ammonia-oxidizing Archaea are associated with different ammonia oxidation uptake rates [65,66], and different ammonia oxidation kinetics reflect different life strategies [65]. Thus, the phylogenetic separation of OTUs with a higher abundance in the microcosms with high CH_4_ concentrations from those preferring low CH_4_ concentrations might indicate the existence of closely related but phylogenetically and functionally distinct *Methylobacter* strains that co-exist in the same environment. In addition to indicating the phylogenetic separation of function, these results also suggest a bias in enrichment and culturing approaches, as most isolates cluster with OTUs that are active at high CH_4_ concentrations.

### 4.2. Temperature Response

Rising temperatures are often affiliated with increases in metabolic activity [67], as shown by the temperature response in growth rates of pure cultures of methanotrophs [37]. In line with this, we did see significant effects of temperature on cellular CH_4_ oxidation rates in *M. tundripaludum* SV96, although the differences were minor at 0.1% CH_4_. In comparison, temperature had small and inconsistent effects on soil CH_4_ oxidation rates and *pmoA* transcript abundances at both 0.1% and 1% CH_4_. Inconsistent temperature effects on CH_4_ oxidation might be caused by several factors, such as changes in cell growth, growth efficiency, cell numbers, cellular protein contents, and types of active MOB strains, all of which may be affected by substrate and nutrient concentrations. In a study of Arctic lakes, CH_4_ oxidation rates were not increasing with increasing temperature when the substrate was limiting [38]. Thus, there might be a physiological response to temperature that, due to CH_4_ limitation, looks like a lack of temperature response. This could be the case in some of our microcosms, particularly at the lowest (0.1%) CH_4_ concentration, possibly explaining the small temperature effect seen at 0.1% CH_4_ in the experiment with *M. tundripaludum* SV96. CH_4_ oxidation has frequently been observed as being less temperature-sensitive than CH_4_ production, but this phenomenon has not yet been explained [35]. Future studies should, therefore, address intracellular physiological responses in MOB at different temperatures and not only quantify CH_4_ oxidation rates and growth. Temperatures from 8 °C to 15 °C do occur in the topsoil on warm summer days in Svalbard [27], and although the community must be physiologically capable of responding to such temperature changes, we are still not aware of how.

Shifts in the active members of the MOB community may also influence the temperature response of a microbial community, but we did not observe any consistent transcriptional shifts among the active members with a change in temperature. Thus, within the temperature range covered in this study, shifts in the transcriptionally active community did not seem to be a significant response.

## 5. Conclusions

We found that MOB in Arctic peat soils from Svalbard respond to changes in CH_4_ concentrations, but the nature of the response depends on whether the peat has been exposed to herbivory. Herbivory “conditioning” simultaneously influenced the presence and activity of different MOB strains, community CH_4_ oxidation, and the effect of increased CH_4_ concentration on CH_4_ oxidation rates. Temperature, on the other hand, had only minor effects on the MOB community and its CH_4_ uptake. However, as CH_4_ production is expected to increase with higher temperatures, we hypothesize that the resulting higher peat soil CH_4_ concentrations will influence MOB activity and CH_4_ oxidation potential, but this shift will be influenced by the extent of the herbivory.

## Data Availability

The data presented in this study are openly available in the European Nucleotide Archive (ENA), project PRJEB48225.

## Supplementary Materials

The following are available online at https://www.mdpi.com/2076-2607/9/10/2080/s1, Figure S1: CH_4_ oxidation rates from Exp II emphasizing the comparison between 0.1 % and 1 % headspace CH_4_ concentrations. Figure S2: CH_4_ oxidation rates from Exp I before and and after the three-week incubation. Figure S3: CH_4_ oxidation rates from Exp II before and and after the three-week incubation. Figure S4: CH_4_ oxidation rates from Exp II emphasizing the comparison between 8 and 15 °C. Figure S5: CH_4_ oxidation rates from Exp I emphasizing the comparison between 8 °C and 15 °C (corrected for dissolved CH_4_). Figure S6: CH_4_ oxidation rates from Exp II emphasizing the comparison between 8 °C and 15 °C (corrected for dissolved CH_4_). Figure S7: CH_4_ oxidation per cell for *Methylobacter tundripaludum* SV96. Table S1: Number of sequences and OTUs at the different stages of the bioinformatic pipeline. Figure S8: Redundancy analysis of the effect of CH_4_ concentration and temperature on the MOB communities after incubation (*pmoA* transcripts using the A189F/A682R primer pair). Figure S9: Distribution of MOB taxa (A189F/A682R primer set) in grazed and exclosed peat soils after incubation at 0.1% CH_4_ and at 1% CH_4_. Figure S10: Relative abundances of *pmoA* transcripts representing MOB OTUs (A189F/A682R primer pair) from grazed peat soil microcosms after incubation at 0.1 % and 1% CH_4_ concentration. Figure S11: Relative abundances of *pmoA* transcripts representing MOB OTUs (A189F/A682R primer pair) from exclosed peat soil microcosms after incubation at 0.1 % and 1% CH_4_ concentration. Figure S12: Phylogenetic representation of the “unconditional bioindicator OTUs” for 0.1% CH_4_ (blue) and 1% CH_4_ (red) incubations, cultivated MOB (bold, italic) and closely related environmental sequences retrieved from the NCBI GenBank
