# Correction: Rainer et al. The Influence of Above-Ground Herbivory on the Response of Arctic Soil Methanotrophs to Increasing CH_4_ Concentrations and Temperatures. *Microorganisms* 2021, *9*, 2080

**DOI:** 10.3390/microorganisms9122535

**Published:** 2021-12-08

**Authors:** Edda M. Rainer, Christophe V. W. Seppey, Caroline Hammer, Mette M. Svenning, Alexander T. Tveit

**Affiliations:** 1 Department of Arctic and Marine Biology, UiT, The Arctic University of Norway, 9037 Tromsø, Norway; seppey@uni-potsdam.de (C.V.W.S.); c.hammer@boku.ac.at (C.H.); mette.svenning@uit.no (M.M.S.); alexander.t.tveit@uit.no (A.T.T.); 2 Institute of Environmental Sciences and Geography, University of Potsdam, Karl-Liebknecht-Str. 24-25, 14476 Potsdam, Germany

The authors wish to make the following corrections to this paper [1]:

**Data Availability Statement**: Reads will be available upon acceptance of the manuscript.

To the correct version, as follows:

**Data Availability Statement**: The data presented in this study are openly available in the European Nucleotide Archive (ENA), project PRJEB48225.

The authors would like to apologize for any inconvenience caused to the readers by these changes.
