# Supplementary material for: The Influence of Above-Ground Herbivory on the Response of Arctic Soil Methanotrophs to Increasing CH4 Concentrations and Temperatures
Source: Microorganisms. 2021 Oct 2;9(10):2080. doi: 10.3390/microorganisms9102080 (PMC8540837; doi:10.3390/microorganisms9102080)
Supplement: Supplementary file 1 [file microorganisms-09-02080-s001.zip › microorganisms-1377728-supplementary.pdf]

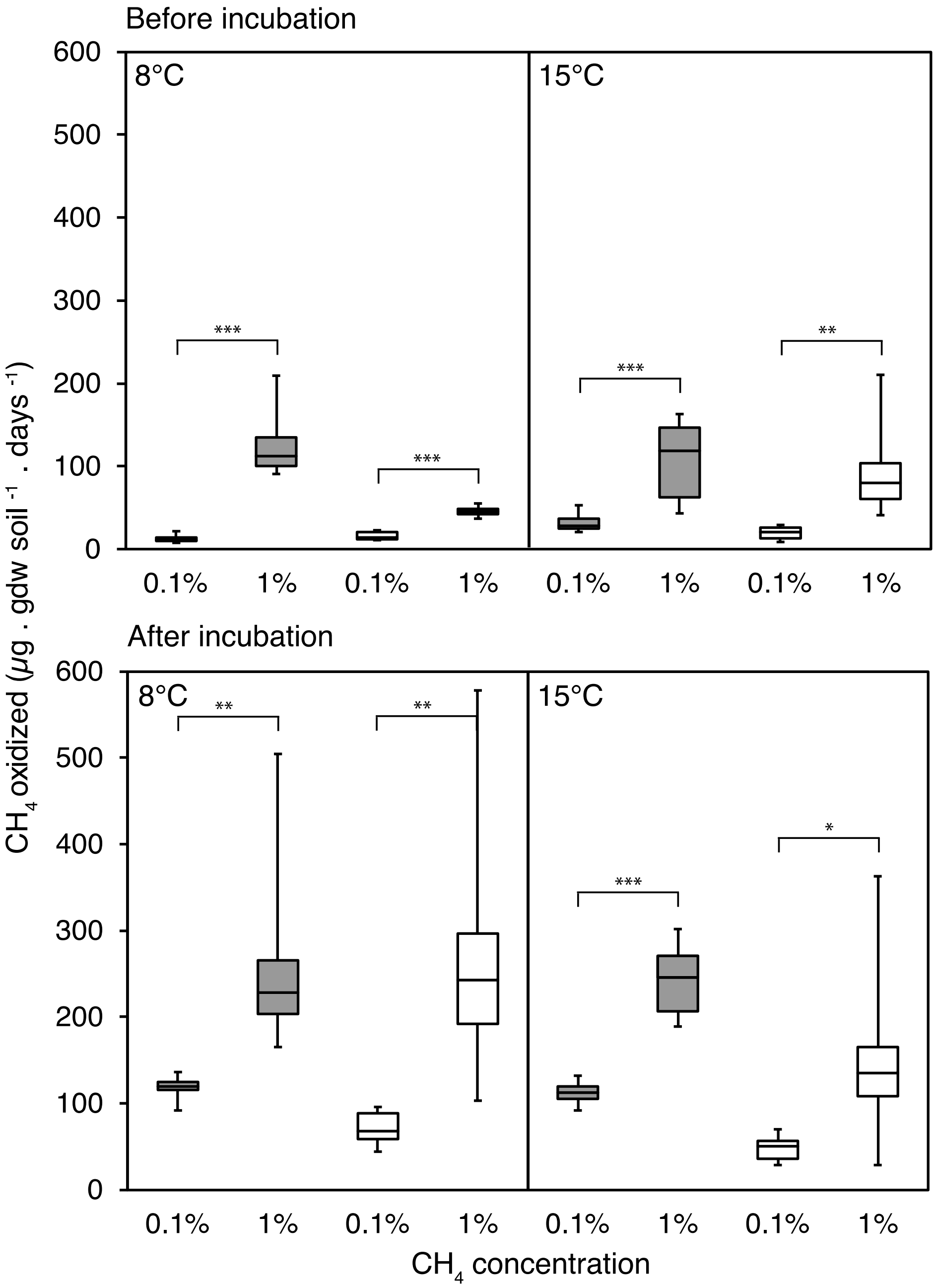

Supplementary Figure S1. CH_4 oxidation rates from Exp II emphasizing the comparison between 0.1% and 1% headspace CH_4 concentrations. Results are shown for measurements collected before (top) and after (bottom) the three-week incubation, and at 8 (left) and 15 °C (right). Grazed microcosms are shown in gray; exclosed microcosms in white. Comparison using linear mixed models with peat blocks as random variables shows significant differences in the CH_4 oxidation rates between CH_4 concentrations (*$p < 0.05$; ** $p < 0.01$; *** $p < 0.001$).

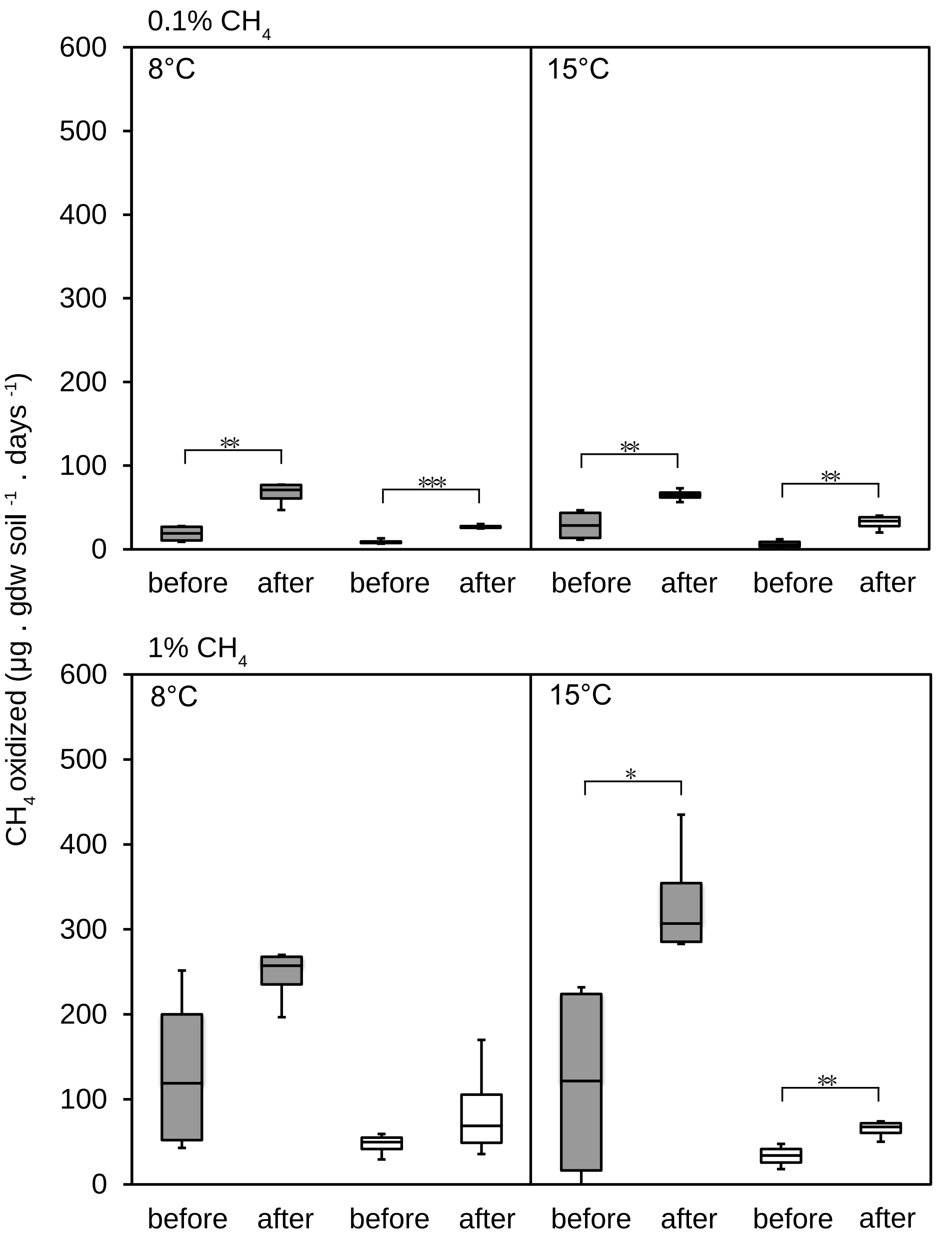

Supplementary Figure S2. CH_4 oxidation rates from Exp I before and and after the three-week incubation, at 8 °C (left) and 15 °C (right) and 0.1% (top) and 1% CH_4 headspace concentrations (bottom). Grazed peat soil microcosms are shown in gray; exclosed peat soil microcosms in white. Comparison using linear mixed models with peat blocks as random variables shows significant differences in the CH_4 oxidation rates before and after the incubation (*$p < 0.05$; ** $p < 0.01$; *** $p < 0.001$).

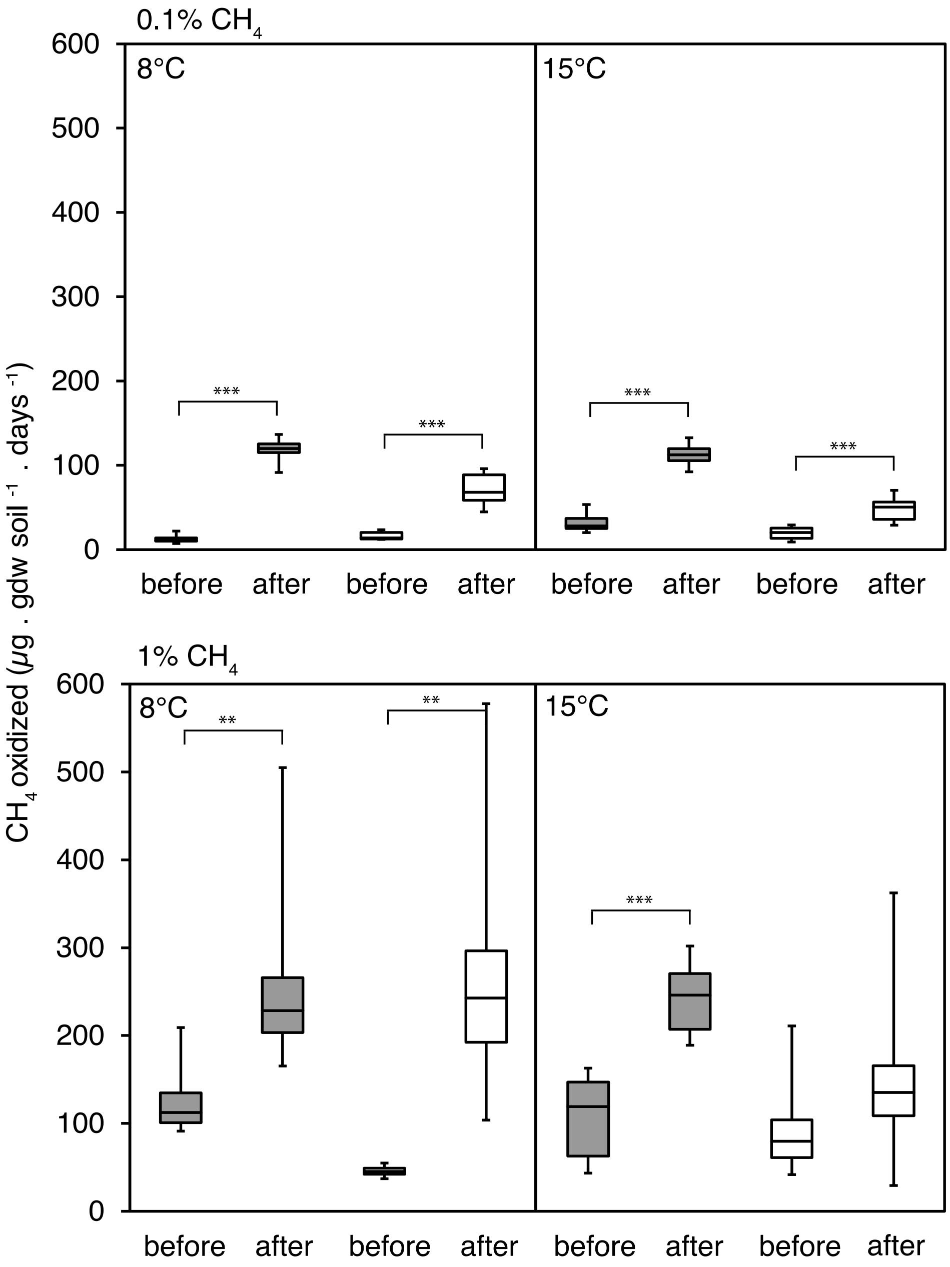

Supplementary Figure S3. CH_4 oxidation rates from Exp II before and and after the three-week incubation at 8°C (left) and 15 °C (right) and 0.1% (top) and 1% CH_4 headspace concentrations (bottom). Grazed peat soil microcosms are shown in gray; exclosed peat soil microcosms in white. Comparison using linear mixed models with peat blocks as random variables shows significant differences in the CH_4 oxidation before and after the incubation (* $p < 0.05$; ** $p < 0.01$; *** $p < 0.001$).

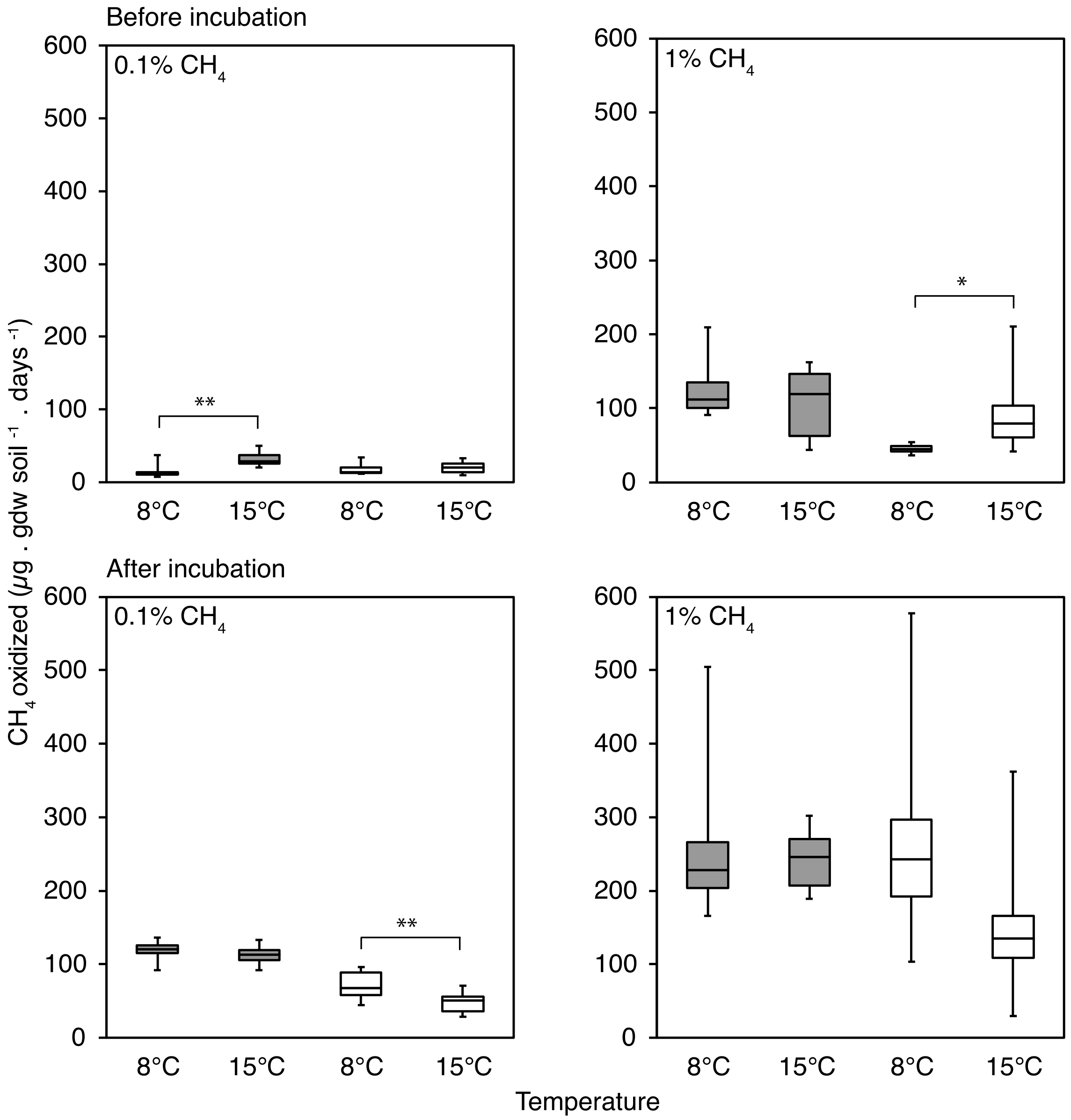

Supplementary Figure S4. CH_4 oxidation rates from Exp II emphasizing the comparison between 8 °C and 15 °C. Results are shown for measurements collected before (top) and after (bottom) the three-week incubation, and at 0.1% (left) and 1% CH_4 (right). Grazed microcosms are shown in gray; exclosed microcosms in white.Comparison using linear mixed models with peat blocks as random variables shows significant differences in the CH_4 oxidation rates between temperatures (* $p < 0.05$; ** $p < 0.01$).

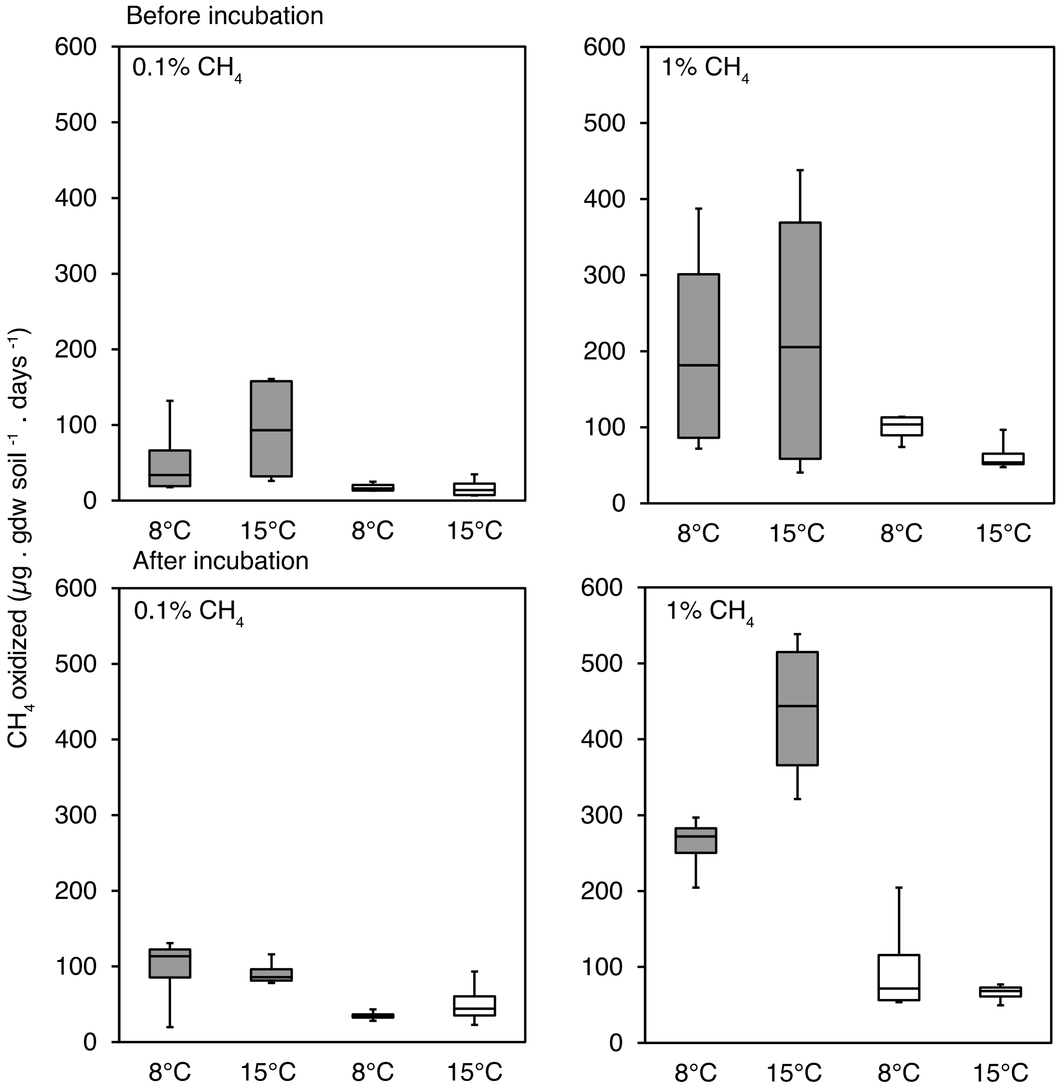

Supplementary Figure S5. CH_4 oxidation rates from Exp I emphasizing the comparison between 8 °C and 15 °C. Results are shown for measurements collected before (top) and after (bottom) the three-week incubation, and at 0.1% (left) and 1% CH_4 (right). The CH_4 oxidation rates presented here are corrected for the different amounts of CH_4 dissolved at 8 °C and 15 °C. Grazed microcosms are shown in gray; exclosed microcosms in white. Comparison using linear mixed models with peat blocks as random variables shows significant differences in the CH_4 oxidation rates between temperatures (* $p < 0.05$).

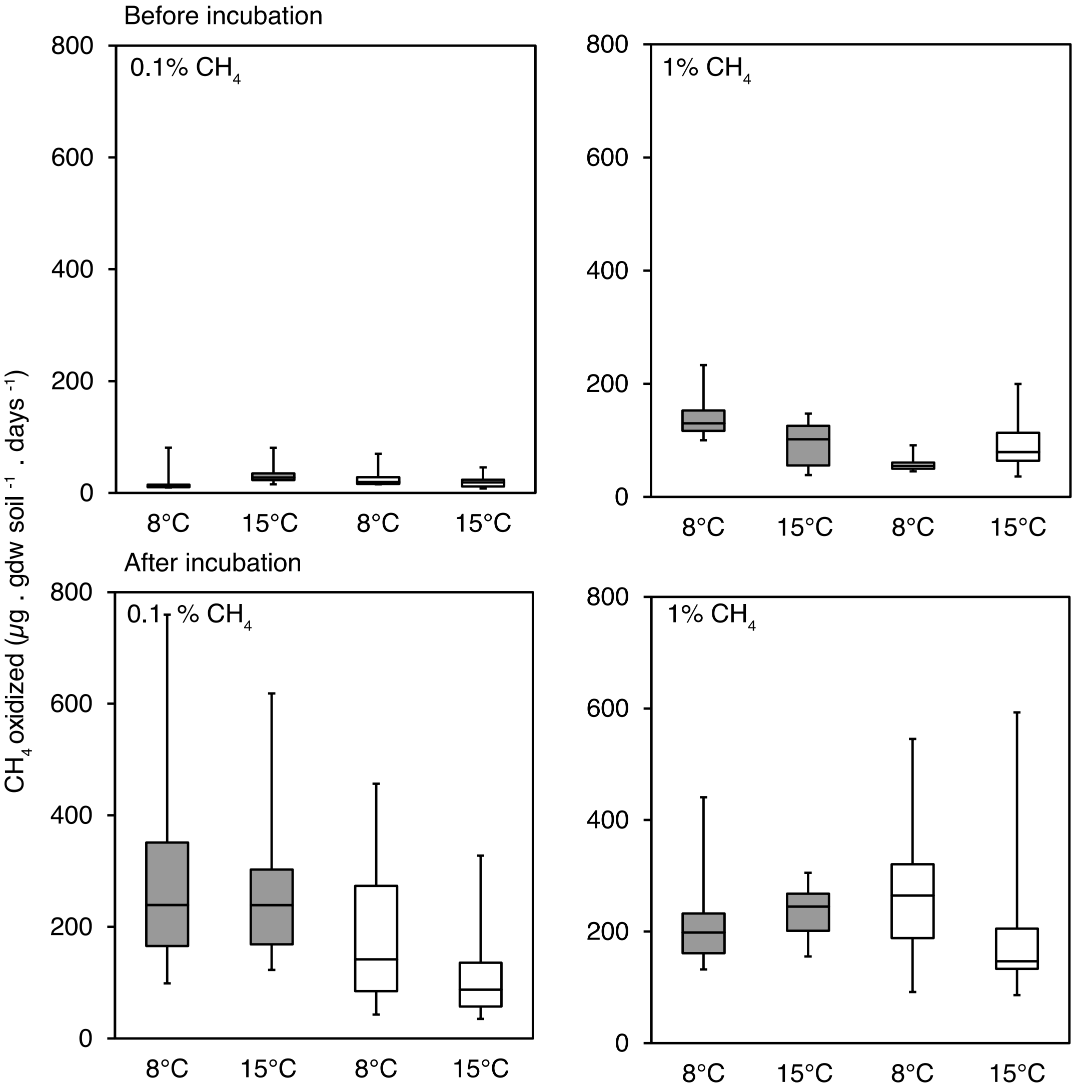

Supplementary Figure S6. CH_4 oxidation rates from Exp II emphasizing the comparison between 8 °C and 15 °C. Results are shown for measurements collected before (top) and after (bottom) the three-week incubation, and at 0.1% (left) and 1% CH_4 (right). The CH_4 oxidation rates presented here are corrected for the different amounts of CH_4 dissolved at 8 °C and 15 °C. Grazed microcosms are shown in gray; exclosed microcosms in white. Comparison using linear mixed models with peat blocks as random variables shows significant differences in the CH_4 oxidation between temperatures (* $p < 0.05$; ** $p < 0.01$).

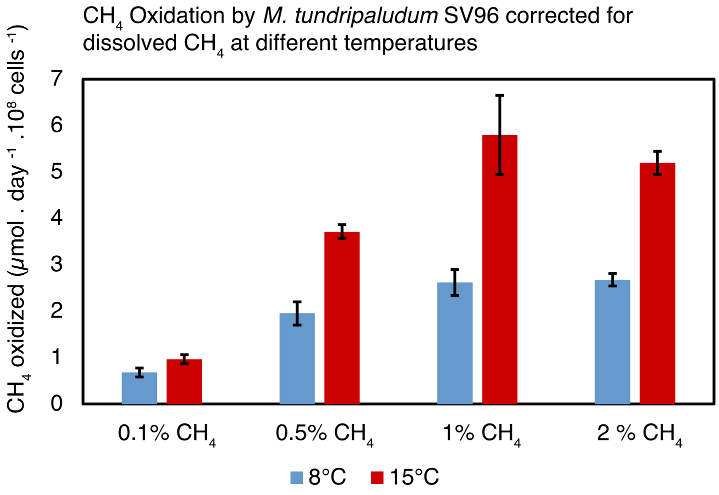

Supplementary Figure S7. CH_4 oxidation per cell for *Methylobacter tundripaludum* SV96 at 8 °C (blue) and 15 °C (red), with CH_4 headspace concentrations ranging from 0.1to 2%. The CH_4 oxidation rates have been corrected for the amount of CH_4 dissolved at 8 °C and 15 °C.

Supplementary Table S1. Number of sequences and OTUs at the different stages of the bioinformatic pipeline.

Pipeline step	Sequences		OTUs	
primer set	mb661	A682	mb661	A682
merging	2068297	1033135	---	---
quality check	1270623	438874	---	---
trimming	1240464	426302	---	---
chimera removal	1162251	422389	---	---
frameshift removal	1096123	394153	---	---
clustering	1096121	394153	11641	5075
length selection	1096106	393547	11631	4952

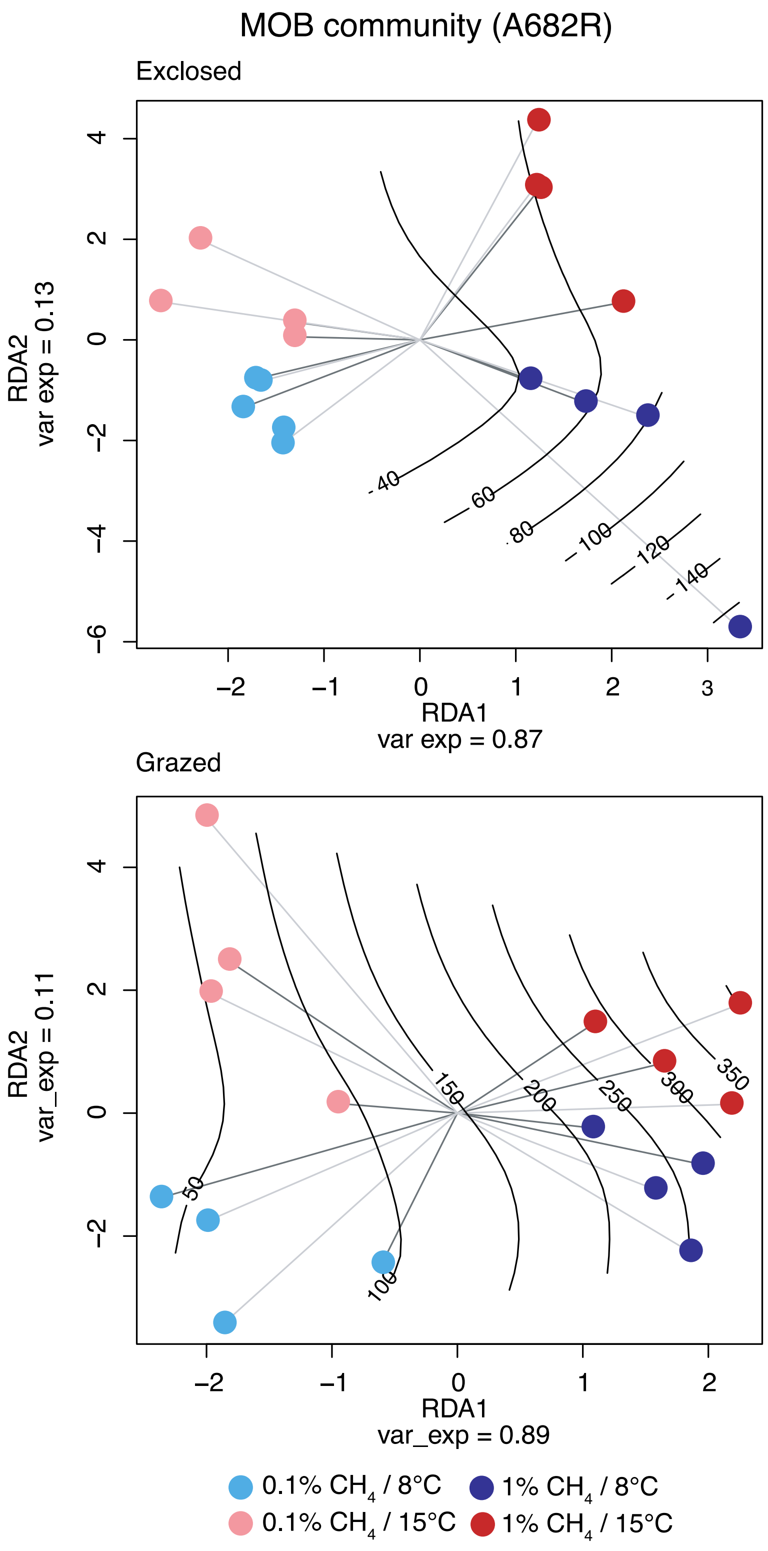

Supplementary Figure S8. Redundancy analysis of the effect of CH_4 concentration and temperature on the MOB communities after incubation (*pmoA* transcripts using the A189F/A682R primer pair). The effect of CH_4 concentration and temperature was corrected according to the peat block replicates (represented by gray shaded lines). Samples are labeled according to CH_4 concentration (light colors — 0.1% CH_4, dark colors — 1%CH_4) and temperature (blue — 8 °C and red — 15 °C). The black lines indicate a projection of the measured CH_4 oxidation rates (µg of CH_4 oxidized per gram dry soil and day).

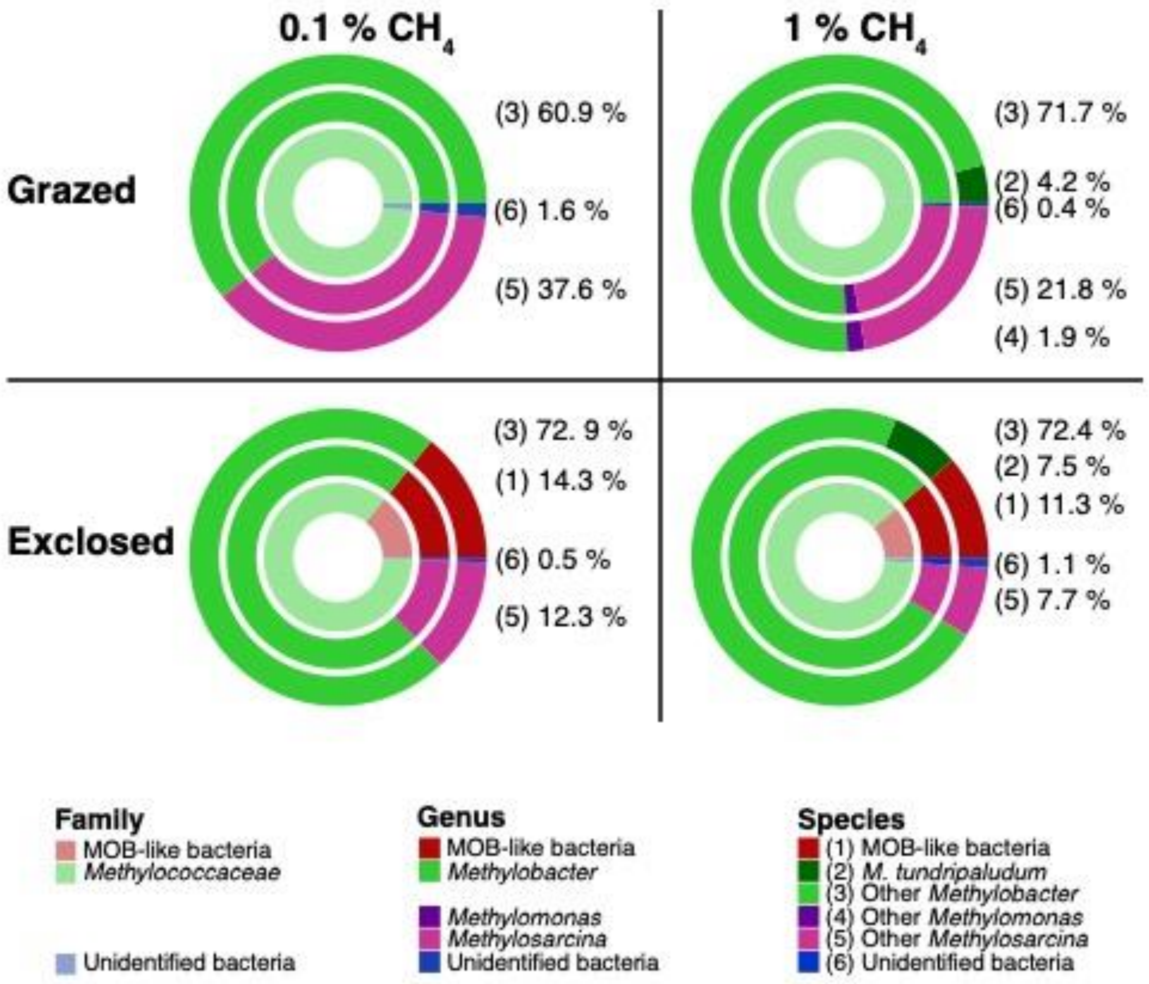

Supplementary Figure S9. Distribution of MOB taxa (A189F/A682R primer set) in grazed and exclosed peat soils after incubation at 0.1% CH_4 and at 1% CH_4 (16 soil samples per concentration and 16 soil samples per grazing condition). As temperature had no significant effect on the MOB community, these 16 samples include both the 8 °C and 15 °C incubations. Taxa with a relative abundance of > 1% are represented at the full taxonomic resolution, whereas those taxa not reaching the 1% threshold were added to their broader taxonomic group, one lvel lower in taxonomic resolution. The different rings represent different taxonomic levels from family to genus to species moving from the innermost ring outward. Relative abundances for the species level are given in %.

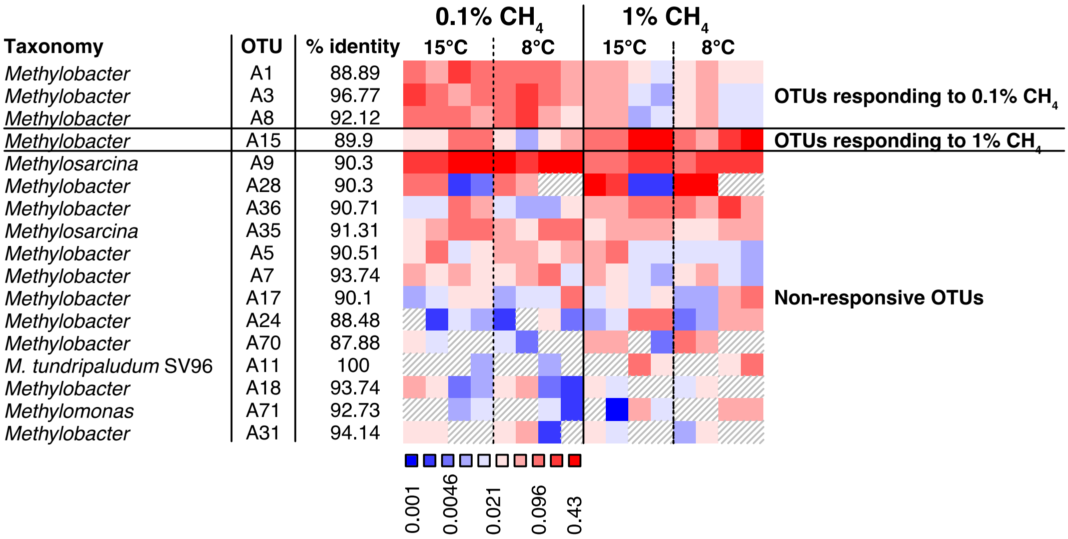

Supplementary Figure S10. Relative abundances of *pmoA* transcripts representing MOB OTUs (A189F/A682R primer pair) from grazed peat soil microcosms after incubation at 0.1% and 1% CH_4 concentrations. OTUs with higher relative abundance at 0.1% CH_4 are shown in the first block (left) and those with higher relative abundance at 1% CH_4 in the second block (right). The non-responsive OTUs with the highest relative abundance are shown in the third block. The number of OTUs included in the figure make up 90% of the total MOB community transcription. OTUs are identified as the letter A (indicating that these are part of the A682R dataset) and a number. The percentage of identity between the OTUs and the best hit in the database is given next to the OTU identification. Relative abundances reach from low abundances (blue) to high (red).

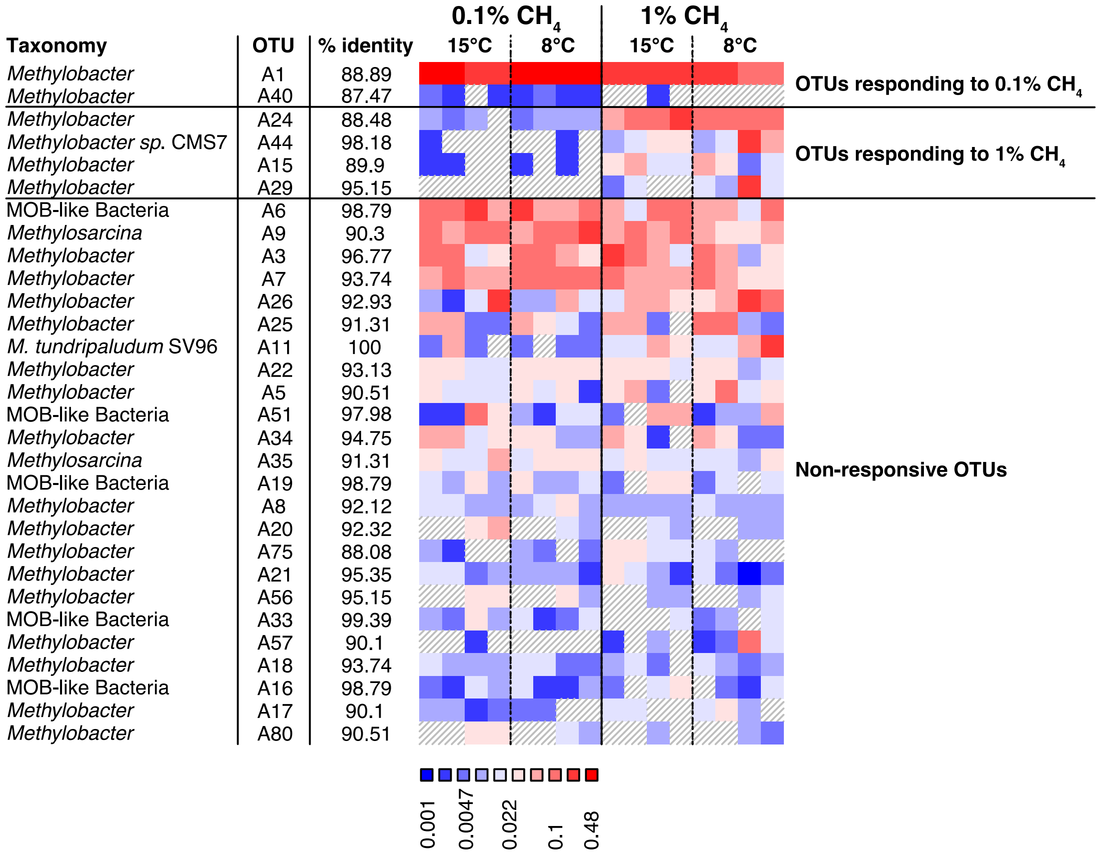

Supplementary Figure S11. Relative abundances of *pmoA* transcripts representing MOB OTUs (A189F/A682R primer pair) from exclosed peat soil microcosms after incubation at 0.1% and 1% CH_4 concentration. OTUs with higher relative abundance at 0.1% CH_4 are shown in the first block (left) and those with higher relative abundance at 1% CH_4 in the second block (right). The non-responsive OTUs with the highest relative abundance are shown in the third block. The number of OTUs included in the figure make up 90% of the total MOB community transcription. OTUs are identified as the letter A (indicating that these are part of the A682R dataset) and a number. The percentage of identity between the OTU and the best hit in the database is given next to the OTU identification. Relative abundances reach from low abundances (blue) to high (red).

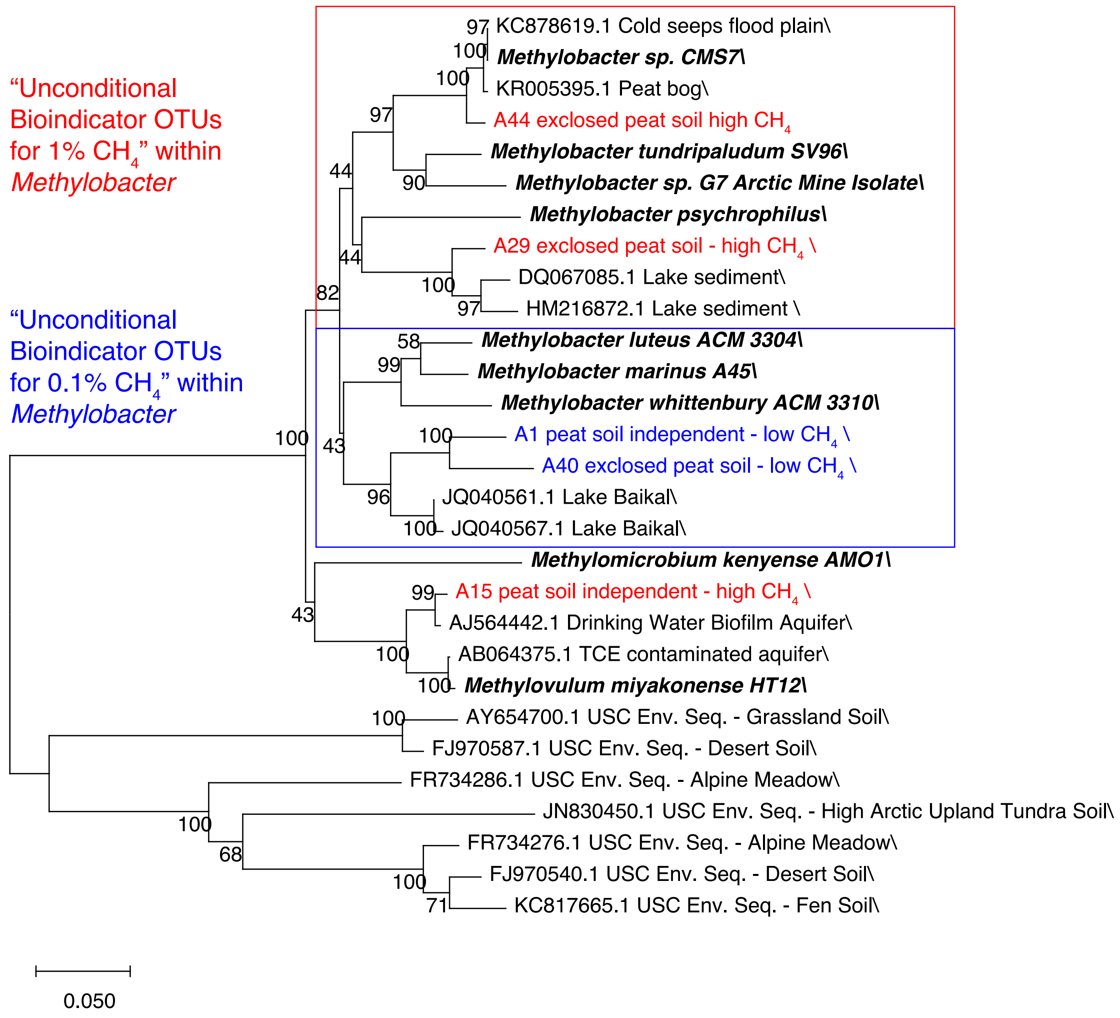

Supplementary Figure S12. Phylogenetic representation of the "unconditional bioindicator OTUs" for 0.1% CH_4 (blue) and 1% CH_4 (red) incubations, cultivated MOB (bold, italic) and closely related environmental sequences retrieved from the NCBI GenBank. All OTUs included were identified as bioindicators both in the grazed and the exclosed peat soil microcosms, or in only one of the soil types, being absent from the other soil type. The first type is called "peat soil independent". The other type of OTUs, which were specifically assigned as bioindicators for one of the peat soil types, are labeled as either grazed or exclosed. The tree is based on a 452 nucleotide alignment, using the neighbor-joining method with the Jukes-cantor correction and 500 bootstraps. The length of the branches is based on a scale of 0.05 changes per nucleotide.
